# Modulation of Proteinoid Electrical Spiking Activity with Magnetic Nanoparticles

**DOI:** 10.1021/acs.langmuir.5c00932

**Published:** 2025-05-30

**Authors:** Panagiotis Mougkogiannis, Andrew Adamatzky

**Affiliations:** Unconventional Computing Laboratory, 1981 University of the West of England, Bristol BS16 1QY, U.K.

## Abstract

This study looks at how proteinoid microspheres and their magnetic polystyrene (PS) hybrids behave electrochemically. It also explores their computational abilities. These systems show complex membrane potential dynamics. Pure proteinoids spike without external influence, ranging from 5.39 to 9.81 mV. In contrast, PS-modified variants exhibit sinusoidal oscillations. Their behavior can be described by the equation *V*(*t*) = *A* sin­(2π*ft*) + *V*_offset_, where *A* is about 1.5 mV and *f* is around 0.05 Hz. Electrochemical impedance spectroscopy shows key differences in charge transport. The PS-modified systems have better conductivity: |*Z*|_PS_ = 7.22 × 10^4^ Ω compared to |*Z*|_prot_ = 2.03 × 10^5^ Ω. The systems can perform Boolean logic operations with a 5 mV threshold. They show time-dependent gate behavior, making them suitable for unconventional computing applications. Doping with Fe­(NO_3_)_3_ changes the electrical response. This happens through redox processes where Fe^3+^ gains an electron to become Fe^2+^. As a result, there are greater potential differences and more complex timing behaviors. These findings help us understand proteinoid-based bioelectricity better. They also show how these building blocks can be used in biomolecular computing systems.

## Graphical abstract

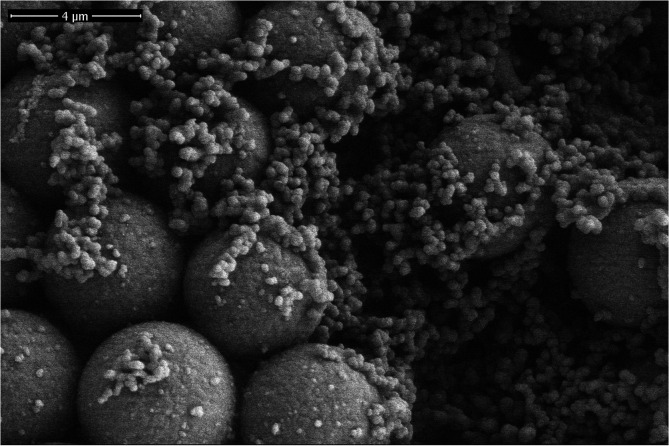

## Introduction

The search for life’s origins has prompted studies of different prebiotic systems, like proteinoids.^1^ Sidney W. Fox discovered proteinoids, or thermal proteins.^2,3^ These are protein-like molecules.^4^ They form abiotically from amino acids when certain conditions are met.^5^ These molecules can self-organize.^6^ They form microspheres that look like primitive cells.^7^ Proteinoids can form cell-like structures and show some catalytic functions.^8^ This makes them a key model for studying early biological processes and the origins of cellular life.^9^

Recent advances in biomolecular computing and magnetic control materials show the promise of these systems for new uses.^7,10–13^ In biomolecular computing, researchers have created DNA and protein systems.^14–18^ These systems can perform complex logic operations. They use the high parallelism and low power needs of biomolecules like DNA and enzymes. DNA machines can process electronic inputs and perform complex calculations. This development is leading to bioelectronic computers that connect with biological systems. These computers have uses in areas like in vivo diagnostics and drug delivery. At the same time, magnetic control biomaterials have advanced a lot, especially in tissue engineering and drug delivery.^19–23^ Magnetic nanoparticles, like iron-doped ones, allow us to control cell behavior and release drugs using external magnetic fields.^24–28^ This is shown in magneto-responsive scaffolds and hyperthermia treatments. These advancements show a rising interest in combining biomolecular systems with external controls like magnetic fields. The goal is to create responsive and programmable biomaterials for computing and biomedical uses.

Recent research highlights the electrical properties of proteinoids.^29^ They can generate membrane potentials and show behavior similar to action potentials.^30^ These findings create exciting chances to develop artificial systems with bioelectric features.^31^ Bioelectricity in synthetic systems, such as proteinoids, offers a special method to explore bioelectric signaling.^32^ This signaling is key to understanding how early life developed.^33^

We’ve made progress in understanding proteinoid electrical activity.^34^ However, we still struggle to control and modulate these signals.^35^ Current methods do not allow for accurate and adaptable manipulation of bioelectric activity in proteinoids.^36^ Creating a way to use magnets to control proteinoid electrical activity would be a big step forward.^37–40^ This method could be noninvasive and easy to adjust, benefiting both research and possible uses. This presents a significant challenge in the development of tunable bioelectric systems.^41,42^

Controlling bioelectric signaling in protein-like materials is difficult. There are several main challenges that make it hard to achieve precision and reproducibility. The varied and changing nature of proteinoid assemblies makes it hard to get consistent electrical responses. Their self-assembled structures show different charge transport properties. Next, adding magnetic responsiveness to these systems without losing their bioelectric function is tricky. It involves solving material compatibility issues. For example, magnetic dopants like iron ions can upset the sensitive balance of molecular interactions that control membrane potential dynamics. Third, scaling these systems for real use, like biomolecular computing or biosensing, needs strong methods. These methods must keep signals stable and responsive in different environments. Tackling these challenges is key to realizing the potential of proteinoid-based systems. These systems can serve as controllable bioelectric platforms. This highlights the need for noninvasive magnetic control strategies.^43–46^

We suggest using iron doping.^47,48^ This will add magnetic properties to proteinoid microspheres.^49,50^ Adding Fe^2+^/Fe^3+^ ions to the proteinoid structure can change membrane properties. It can also create sites that interact with external magnetic fields. Magnetic fields might affect iron-doped proteinoid systems in several ways. They could change how ions move through channels. They might also alter the membrane’s potential and adjust the shape of the proteinoids. We need more research to understand these mechanisms and determine if magnetic control can work.^51^

Adding polystyrene (PS) microparticles (*d* = 5.0 ± 0.5 μm) to proteinoid systems creates a strong platform. This setup helps us study magnetically controlled behavior. PS microspheres act as size-controlled templates for proteinoid assembly. This setup helps us study the effects of iron doping systematically. It also keeps the bioelectric properties of the proteinoid intact. Past studies showed that we can create shape-tunable macroparticles using PS cores.^52,53^ Also, methods for making crystalline PS nanoparticles^54^ guided our surface modification strategy. Biopolymer microencapsulation techniques^55^ and PS-proteinoid hybrid formation work together. This combination allows for the controlled assembly of structures that respond to magnets.

This study aims to:

- Synthesize and characterize iron-doped proteinoid microspheres.
- Investigate their spontaneous electrical activity.
- Show magnetic field control of action potential-like signals.
- Understand the mechanism of magnetic modulation.

Developing magnetically controlled proteinoid microspheres could impact many fields.^56,57^ It would give insights into bioelectricity in early systems. Also, it would create a new way to study the evolution of bioelectric signaling. It would also lead to controllable biomimetic materials.^58^ These materials can be used in synthetic biology, drug delivery with magnets, and biosensing.^59^

## Experimental Section

### Materials

All chemicals were purchased from Sigma-Aldrich and used without further purification: magnetic polystyrene microspheres (diameter 5.0 ± 0.5 μm), l-glutamic acid (*M*_w_ = 147.13 g, mol^–1^, CAS: 56–86–0), l-aspartic acid (*M*_w_ = 133.10 g, mol^–1^, CAS: 56–84–8), l-phenylalanine (*M*_w_ = 165.19 g, mol^–1^, CAS: 63–91–2), and poly­(d,l-lactic acid) (PDLLA, IV 0.5 dL, g^–1^, CAS: 26023–30–3).

### Synthesis of Magnetic Polystyrene-Proteinoid Composites

The proteinoid synthesis followed a thermal condensation procedure. Equimolar amounts of l-glutamic acid, l-aspartic acid, and l-phenylalanine (each 1.0 g) were combined with PLLA (1.0 g) in a round-bottom flask equipped with a reflux condenser. The mixture was heated to 180 °C under continuous stirring at 150 rpm for 3 h to form a homogeneous melt.

The resulting viscous slurry was dissolved in boiling deionized water (50 mL) under vigorous stirring. The solution was maintained at 100 °C for 30 min to ensure complete dissolution. The hot solution was then rapidly cooled to 4 °C to induce proteinoid self-assembly.

Adding poly­(d,l-lactic acid) (PDLLA) to proteinoid synthesis helps control microsphere formation and stability better. During thermal condensation at 180 °C, PDLLA (*M*_w_ = 150,000 g·mol^–1^) forms bonds with amino acid monomers like Glu, Phe, and Asp. This happens through hydrogen bonding and hydrophobic interactions. This interaction affects self-assembly. It results in more uniform microsphere sizes and stronger structures.

The presence of PDLLA affects the proteinoid formation mechanism through several pathways. The polymer chains serve as templates during cooling. They create nucleation sites for ordered proteinoid aggregation. The amphiphilic nature of PDLLA stabilizes the interface between hydrophilic amino acids and hydrophobic areas. This leads to microspheres with a consistent shape and smaller size variation.

When magnetic PS cores are used, PDLLA-modified proteinoids show better coating uniformity and adhesion. The PDLLA has a polyester backbone. This structure creates compatible interaction sites for the PS surface and the developing proteinoid network. This three-part system–PS–PDLLA–proteinoid—shows better structural stability. It also keeps the desired bioelectric and magnetic response properties. These hybrid microspheres are now more uniform. This change allows for better study of how magnetically controlled action potentials are generated and spread. To form PS-proteinoid hybrids, we mixed 0.5 g of magnetic polystyrene microspheres into the hot proteinoid solution and cooled it down. After sonication for 5 min to ensure uniform dispersion, the mixture underwent the same cooling protocol.

### Product Recovery

We collected the microspheres by centrifugation at 5000 rpm for 10 min. Then, we washed them three times with cold deionized water. Finally, we lyophilized the microspheres for 24 h at −50 °C and 0.001 mbar to get a free-flowing powder. The final product was stored in a desiccator at room temperature until further use.

### Electrical Measurements

We used a high-precision data acquisition system (PICOLOG) to characterize the electrical properties of the proteinoid and proteinoid-PS systems. The system operated at a sampling frequency of *f*_s_ = 1 Hz. The experimental setup employed platinum/iridium (Pt/Ir) electrodes with diameter *d* = 0.1 mm. The working area (*A*) of the needle-like cylindrical electrode was calculated as

1 $$A=\pi r^{2}=\pi {\left(0.05\;\mathrm{mm}\right)}^{2}=7.85\times 10^{-3}\;{\mathrm{mm}}^{2}$$

The electrodes were positioned with a fixed separation distance of *l* = 10 mm in the aqueous suspension to ensure consistent field distribution. This setup allowed us to monitor membrane potential changes over time. It also reduced the effects of electrode polarization.

We calibrated the measurement system with standard electrolyte solutions.^60^ We also validated it before the experiments. All recordings were performed at room temperature (*T* = 298 K) under controlled ambient conditions to ensure reproducibility. We optimized the data acquisition settings. This lets us capture quick potential changes and slow baseline drift.

We used MATLAB and Origin Pro 2023b to process and analyze signals. This helped us extract important parameters like spike amplitude, frequency, and timing patterns. The setup measured time well enough to capture the dynamic behavior of both pure proteinoid and PS-modified systems. It also kept a high signal-to-noise ratio during long recording periods.

Electrochemical characterization was performed using a PalmSense4 potentiostat/galvanostat (PalmSense, U.K.). Cyclic voltammetry (CV) was done at a scan rate of 100 mV, s^–1^. The potential window ranged from −4 V to +4 V, using a Pt/Ir reference electrode and Au screen printed Au electrode. We performed electrochemical impedance spectroscopy (EIS) with a 10 mV AC perturbation on a 0.1 V DC bias. The frequency range spanned from 10^–5^ to 10^6^ Hz with 135 points logarithmically distributed (12.2 points per decade). All measurements were performed at room temperature (*T* = 298 K) in a three-electrode configuration.

## Results and Discussion

### Morphological Analysis of Polystyrene Magnetic Microspheres and Nano-Proteinoid Assemblies

We examined the structure of the synthesized systems using scanning electron microscopy. We optimized the imaging conditions for polymer materials that are sensitive to electron beam. Figure 1 reveals the hierarchical organization of the proteinoid-polystyrene hybrid structures (with a high voltage of 1.00 kV and a pressure of 3.05 × 10^–5^ Torr). Pure polystyrene microspheres (Figure 1a) are very uniform and have a smooth, round shape. Their diameter is about 4.7 μm. This indicates that the synthesis conditions were well-controlled. Magnetic components and proteinoid structures (Figure 1b) create surface roughness. They also cause localized coalescence at interfaces. The microspheres keep their diameter at *d* = 4.676 μm. Higher magnification (15,000×) reveals the detailed texture from effective surface modification. The Glu:Phe:Asp:PDLLA copolymer proteinoids have a unique structure (Figure 1c). They have primary spherical particles that are 0.277 μm in size. These particles are surrounded by secondary nanostructured aggregates. The surface morphology suggests self-assembly processes operating at multiple length scales. Upon introduction of Fe­(NO_3_)_3_, significant morphological changes emerge, as shown in Figure 2. Pyramidal PDLLA structures stand out. They have a base length of 21.6 μm. These structures are decorated with proteinoid microspheres modified with ferrous nitrate. These microspheres have a diameter of 1.094 μm and are larger than unmodified proteinoids (Figure 2a,b). The medium-sized pyramidal structures, measuring 10.77 μm, have a smooth surface. This surface is covered by proteinoid assemblies (Figure 2c). A larger view shows size distributions from 0.5 to 1.190 μm, with organized patterns (Figure 2d). We optimized the imaging parameters for all samples. The settings were HV = 2.00 kV, WD = 5.2–5.3 mm, and *P* = 4.71–7.56 × 10^–6^ Torr. This reduced charging effects and ensured clear resolution for analyzing complex hybrid structures.

**Figure 1:**
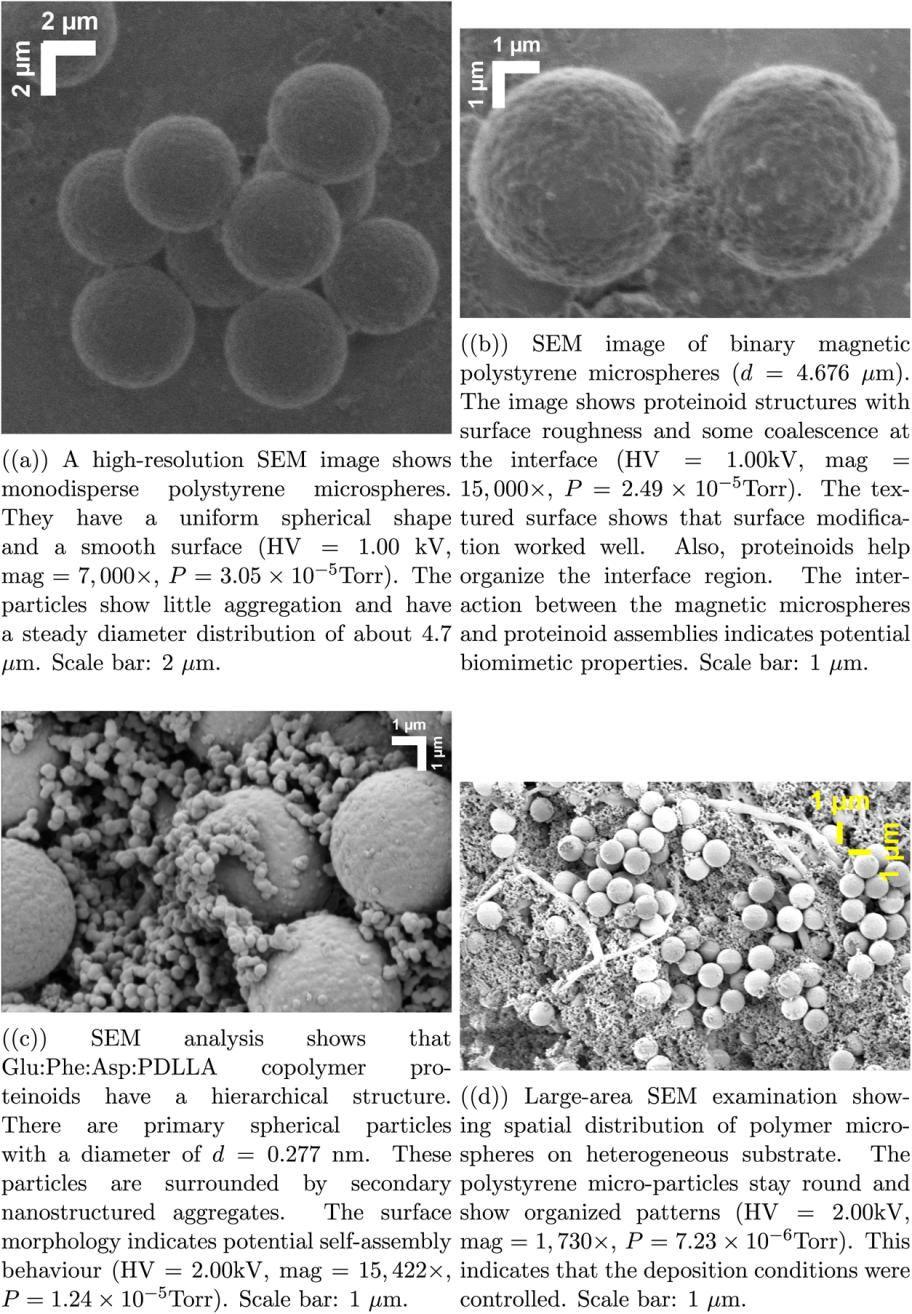
SEM characterization of polystyrene-proteinoid microsphere systems at different magnifications and conditions. All images were taken with an ETD detector. The working distance was kept constant at 8.7 mm. We optimized spot sizes between 1.0 and 3.0 and adjusted beam parameters for imaging polymer specimens.

**Figure 2:**
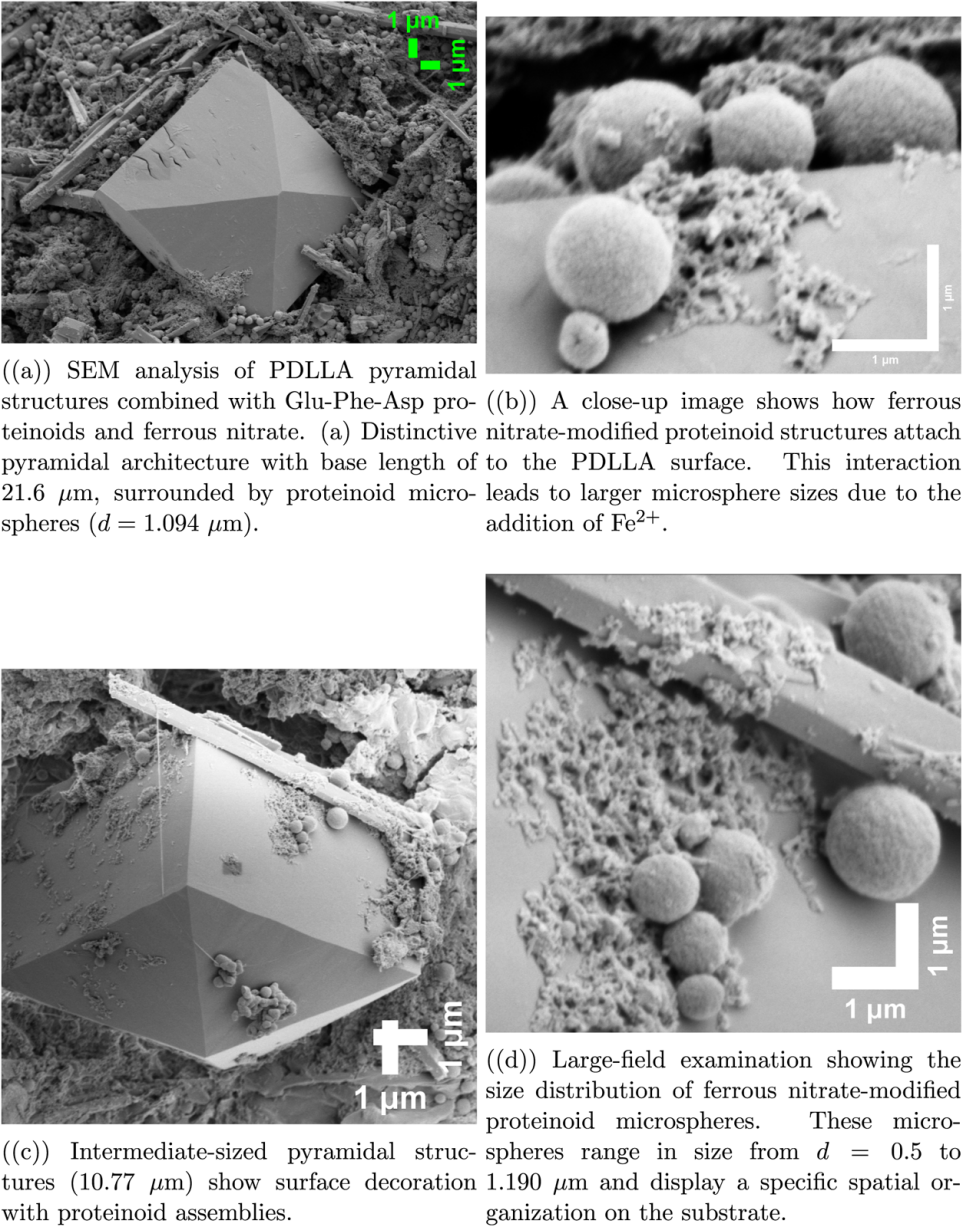
SEM micrographs show PDLLA pyramidal crystals and proteinoid microspheres. These images were taken with an ETD detector at HV = 2.00 kV and WD = 5.2–5.3 mm. The conditions were high vacuum, ranging from 4.71 × 10^–6^ to 7.56 × 10^–6^ Torr. Different magnifications (3000–36,130×) show a clear structure. Images collected with spot size 3.0 and optimized beam parameters to minimize charging effects. Scale bars: 10 μm (a, c), 1 μm (b, d).

Classical nucleation theory^61^ helps explain how proteinoid-PS systems change in shape over time. Spherical proteinoid structures form through homogeneous nucleation. The Gibbs free energy change (Δ*G*) for forming the nucleus is^62^

2 $$\Delta G=-\frac{4\pi r^{3}}{3}\Delta G_{v}+4\pi r^{2}\gamma$$

where *r* is the nucleus radius, Δ*G*_v_ is the volume free energy change, and γ is the surface tension. The observed uniform size distribution (*d* = 4.676 μm) suggests a critical nucleus radius (*r*_c_) that minimizes Δ*G*

3 $$r_{c}=\frac{2\gamma }{\Delta G_{v}}$$

The introduction of Fe^2+^ ions modifies the nucleation process through heterogeneous nucleation, where the contact angle (θ) between the nucleating phase and substrate reduces the energy barrier^63,64^

4 $$\Delta G_{\mathrm{het}}=\Delta G_{\mathrm{hom}}f\left(\theta \right)=\Delta G_{\mathrm{hom}}\frac{(2+\cos\theta ){\left(1-\cos\theta \right)}^{2}}{4}$$

This shows why larger proteinoid microspheres (1.094 μm) occur in Fe­(NO_3_)_3_-modified systems. The lower energy barrier helps them grow. The pyramidal PDLLA structures (*l* = 21.6 μm) likely form through secondary nucleation,^65^ where the growth rate (*G*) follows

5 $$G=A\exp\left(-\frac{\Delta G^{*}}{\mathrm{kT}}\right)\exp\left(-\frac{\Delta E_{D}}{\mathrm{kT}}\right)$$

where ΔG* is the nucleation barrier, Δ*E*_D_ is the activation energy for diffusion, *k* is Boltzmann’s constant, and *T* is temperature. The patterns seen indicate that Ostwald ripening affects late growth stages. In this process, larger structures grow while smaller ones shrink. This happens according to

6 $$\frac{dr}{dt}=\frac{D\gamma V_{m}}{\mathrm{RT}}\left(\frac{1}{r_{c}}-\frac{1}{r}\right)$$

where *D* is the diffusion coefficient, *V*_m_ is the molar volume, *R* is the gas constant, and *t* is time. The hierarchical structures we see in our proteinoid-PS systems resemble natural biomineralization processes.^66^ Classical mineralization happens when ions add one by one to a crystal face. However, our systems show signs of nonclassical pathways, especially with Fe^2+^ ions present.

Proteinoid assemblies act like biomineralization proteins. In these assemblies, acidic residues (α-Glu, β-Asp) help control local supersaturation^67^ (σ). This mimics natural processes like nacre formation.^68^ In nacre, special proteins regulate how minerals deposit. Our system forms ordered structures like those in mollusk shells or bones.^69^ This suggests a biomimetic process at work. The polystyrene matrix serves as an organic template. It works like α-collagen, which guides hydroxyapatite crystal growth in bone tissue. The acidic amino acids in our proteinoid structures probably have roles like those of the acidic proteins in nacre. These proteins help control nucleation and crystal growth using specific ion-binding domains (δ). The hierarchical organization we see stretches from nanoscale (0.277 μm) to microscale (21.6 μm). This mirrors the multilevel structural control found in biological systems. Our Fe^2+^-modified proteinoids group around larger structures like mineral platelets in nacre. In both cases, organic matrices control spacing (Δ*x*) and orientation (θ).

This biomimetic method shows how we control crystal shape and orientation in our Fe^2+^-modified systems. Fe^2+^ ions seem to boost this control. They likely bind to the carboxylate groups (COO^–^) of acidic amino acids. This is like how Ca^2+^-binding proteins regulate biomineralization in nature.

### Spontaneous Membrane Potential Oscillations in Pure Proteinoid and Proteinoid-PS Microspheres

The electrical activity shows clear differences between pure proteinoid and proteinoid-PS microspheres in water. Pure proteinoid systems (Figure 3) show strong spiking behavior with clear traits. The temporal evolution shows steady oscillations lasting up to 2 × 10^5^ s. Spike amplitudes vary from 5 to 30 mV (see Figure 3a). Higher temporal resolution shows complex activity patterns at various time scales (Figure 3b–d).

**Figure 3:**
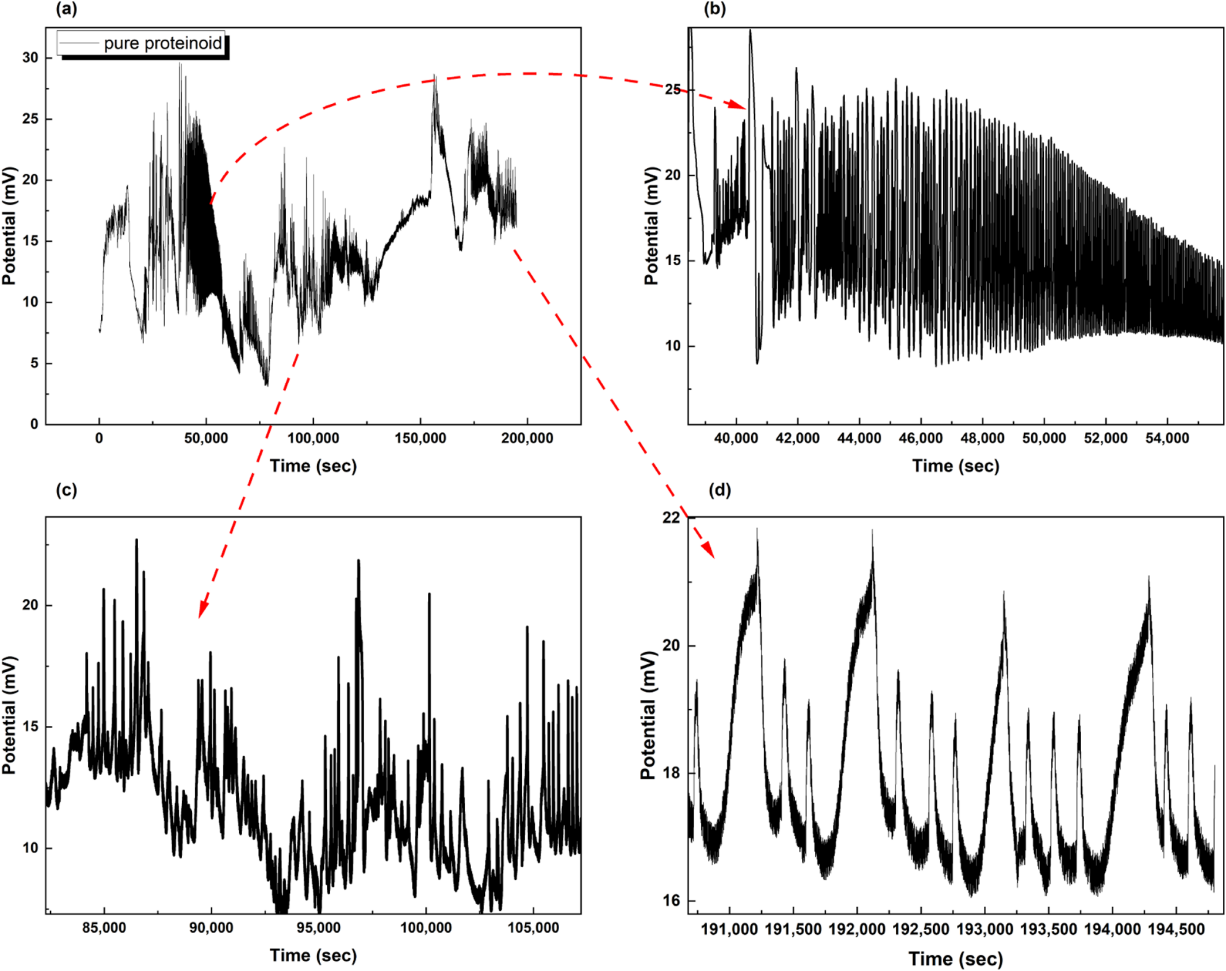
Spontaneous electrical activity recorded from Glu:Phe:Asp:PDLLA proteinoid systems at 1 Hz sampling rate. (a) The membrane potential changed over 2 × 10^5^ s. It showed steady spiking between 5 and 30 mV. (b) A closer look at the period from 40,000 to 55,000 s showed fast oscillations and regular amplitude changes. (c) During 85,000 to 105,000 s, we saw clear spike patterns with amplitudes of 10–20 mV. (d) In the late stage, from 191,000 to 194,500 s, we saw clear periodic spikes. These spikes had a peak-to-peak amplitude of around 5 mV. The baseline recovery remained steady. The multiscale temporal analysis reveals self-sustained electrochemical oscillations characteristic of biomimetic membrane systems.

In contrast, proteinoid-PS microspheres (Figure 4) show modified electrical behavior. The potential evolution starts with a drop to −8 mV. Then, it gradually recovers to −3 mV over 75,000 s (Figure 4a). A close look shows small oscillations with an amplitude of about 0.8 mV (Figure 4b).

**Figure 4:**
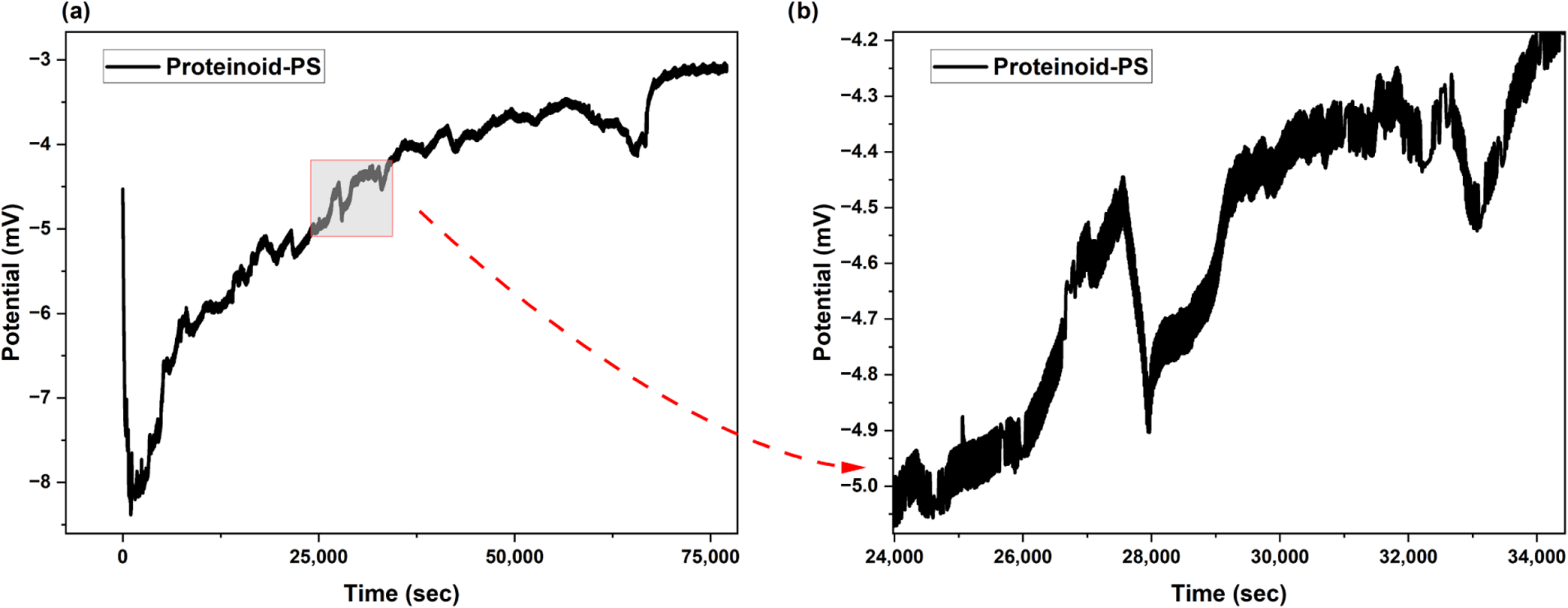
Time-dependent potential measurements of proteinoid-polystyrene (PS) hybrid microspheres. (a) Long-term potential evolution over 75,000 s showing initial rapid decrease to −8 mV followed by gradual recovery and stabilization at −3 mV. The overall trend demonstrates membrane potential development and system equilibration. (b) A closer look at the possible changes between 24,000 and 34,000 s (shown in panel b) shows small oscillations. The amplitude is about 0.8 mV, ranging from −5.0 to −4.2 mV. You can also see distinct step-like patterns. These variations hint at active ion transport across the hybrid proteinoid-PS interface. This might suggest that transient channels or pores form within the microsphere structure. The ongoing oscillations and positive potential drift show that charge separation is happening in the hybrid system.

The key parameters characterizing the spiking behavior can be defined as

7 $$V_{\mathrm{spike}}=V_{\mathrm{peak}}-V_{\mathrm{baseline}}$$

where *V*_spike_ is the spike amplitude, *V*_peak_ is the maximum potential, and *V*_baseline_ is the resting potential. The spike period (*T*) is given by

8 $$T=t_{n+1}-t_{n}$$

where *t*_*n*_ and *t*_*n*+1_ are the times of consecutive spike peaks. The spike frequency (*f*) is calculated as

9 $$f=\frac{1}{T}=\frac{1}{t_{n+1}-t_{n}}$$

The spike duration (τ) is defined as

10 $$\tau =t_{\mathrm{end}}-t_{\mathrm{start}}$$

where *t*_start_ and *t*_end_ mark the beginning and end of a single spike event at half-maximum amplitude. The rate of potential change during a spike can be expressed as

11 $$\frac{dV}{dt}=\frac{V_{\mathrm{peak}}-V_{\mathrm{baseline}}}{\tau_{\mathrm{rise}}}$$

where τ_rise_ is the rise time from baseline to peak. For pure proteinoids, the key values were *V*_spike_ = 5.39–9.81 mV, *T* = 2489–2826 s, and τ = 180–250 s. In contrast, the proteinoid-PS system showed different behavior. It had smaller oscillations and a longer-term potential drift.

The morphological characteristics of the electrical signals can be quantified through several shape parameters

12 $$\mathrm{asymmetry}\;\mathrm{ratio}=\frac{\tau_{\mathrm{rise}}}{\tau_{\mathrm{decay}}}=\frac{t_{\mathrm{peak}}-t_{\mathrm{start}}}{t_{\mathrm{end}}-t_{\mathrm{peak}}}$$

where τ_rise_ and τ_decay_ represent the rise and decay times, respectively. The spike shape factor (SSF) can be defined as

13 $$\mathrm{SSF}=\frac{V_{\mathrm{spike}}}{\tau_{\mathrm{width}}}\cdot \frac{\tau_{\mathrm{rise}}}{\tau_{\mathrm{decay}}}$$

where τ_width_ is the full width at half-maximum. Signal sharpness is characterized by

14 $$\mathrm{sharpness}=\max\left|\frac{d^{2}V}{dt^{2}}\right|$$

For pure proteinoids (Figure 3), the spikes show a unique shape. They rise quickly, with a time constant of about 45 s. Then, they decay more slowly, taking around 135 s. This creates an asymmetry ratio of roughly 0.33. The spikes show consistent shape factors (SSF = 0.021–0.025) and distinct sharp peaks. The proteinoid-PS system (Figure 4) shows smoother potential changes. It has less sharpness and longer transition times. This suggests that the charge transport mechanisms at the interface are different.

### Influence of Fe­(NO_3_)_3_ (0.1 M) on Membrane Potential Dynamics of Proteinoid Systems

Adding Fe­(NO_3_)_2_ changes the electrical activity patterns in pure proteinoid and proteinoid-PS systems. In pure proteinoids (Figure 3), the baseline spiking shows key parameters from eq 7. Here, *V*_spike_ ranges from 5.39 to 9.81 mV. The periodicity, as defined in eq 8, is consistent at *T* = 2489 to 2826 s. The spike duration, τ (see eq 10), is between 180 and 250 s. It has an asymmetry ratio of about 0.33 (refer to eq 12).

Upon Fe^3+^ addition (Figure 5), the dynamics undergo significant modification. The spike amplitude increases to 2–10 mV with irregular fluctuations, while the period *T* becomes less regular. The spike duration τ decreases to 60–120 s, accompanied by a sharper rise time. The potential change rate (see eq 11) rises by about 50%. This shows improved charge transport kinetics.

**Figure 5:**
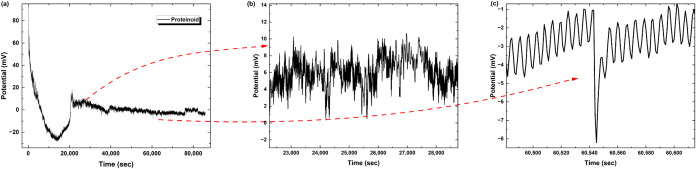
Temporal evolution of membrane potential in proteinoid microspheres after Fe­(NO_3_)_3_ 0.1 M addition (5 mL). (a) A long-term recording shows a quick drop to −20 mV, then recovery and stabilization around 0 mV over 80,000 s. (b) A closer look from 23,000 to 28,000 s reveals irregular shifts with amplitudes between 2 and 10 mV. (c) In a high-resolution analysis from 60,500 to 60,600 s, we see regular oscillations with peak-to-peak amplitudes of about 3 mV. There’s also a sharp hyperpolarization event at 60,550 s that hits −8 mV. The multiscale analysis shows that iron ions change the spiking behavior of proteinoid microspheres. This suggests that iron affects how membrane charge transport works.

The proteinoid-PS system reacts strongly when Fe^3+^ is added (see Figure 6). The initial response shows a dramatic increase in *V*_spike_ to 250 mV, with d*V*/d*t* reaching approximately 5 mV/s. The asymmetry ratio decreases to 0.1, indicating highly asymmetric spikes. This shows a significant change from the non-Fe proteinoid-PS baseline (Figure 4). In that case, oscillations were restricted to 0.8 mV.

**Figure 6:**
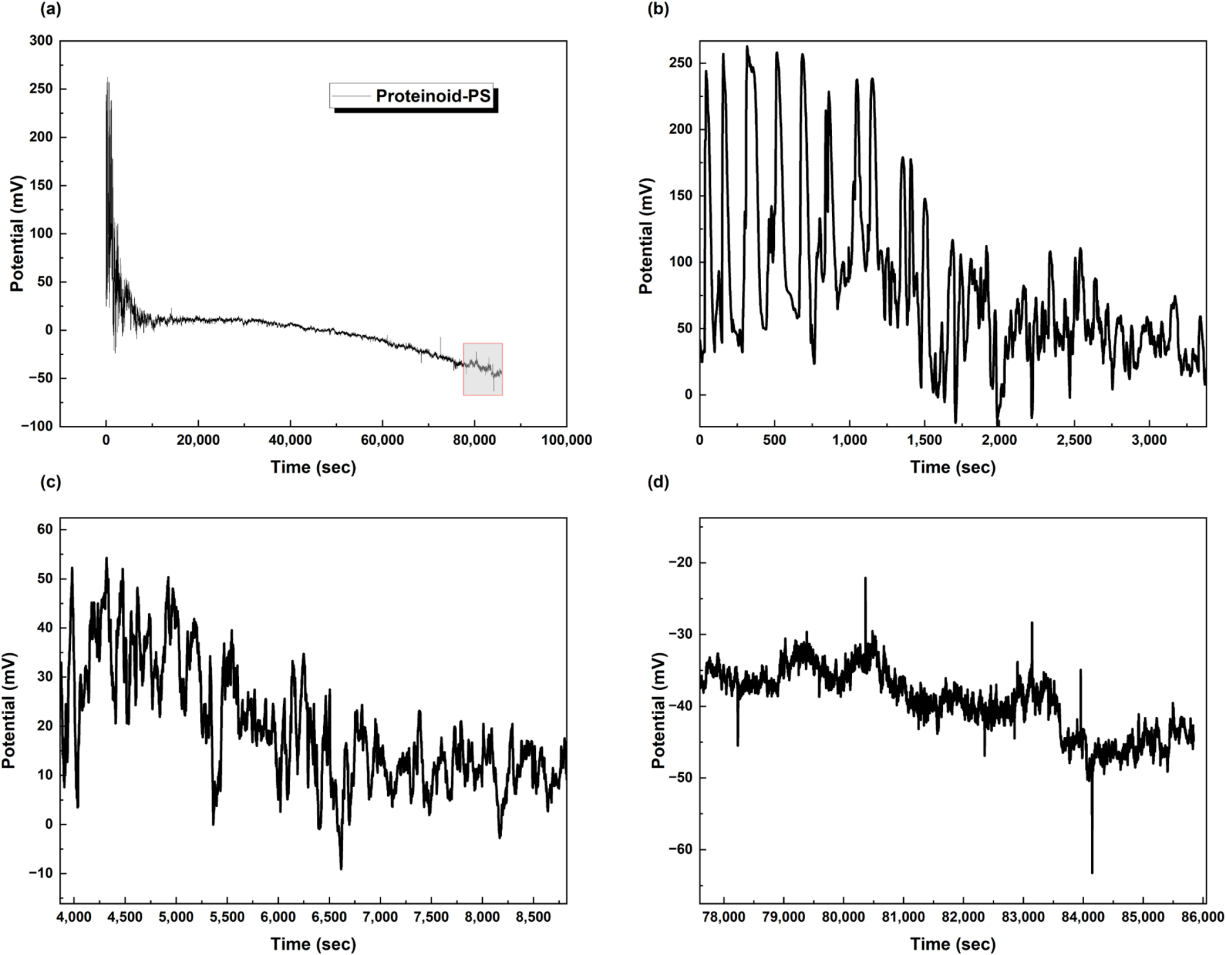
Membrane potential changes in proteinoid-PS microspheres after adding 5 mL of 0.1 M Fe­(NO_3_)_3_ show different behavior than pure proteinoids. (a) Long-term recording over 100,000 s shows a sharp depolarization spike at +250 mV. This is followed by a gradual hyperpolarization to −50 mV. This differs from pure proteinoids, which hyperpolarize to −20 mV. (b) In the early stage (0–3500 s), there are high-amplitude oscillations between 0 and 250 mV with irregular periodicity. (c) The intermediate phase (4000–9000 s) shows dampened oscillations from 0 to 50 mV and decreasing amplitude. (d) In the late stage (78,000–86,000 s), the behavior is stable, with a negative potential ranging from −30 to −50 mV and small fluctuations. The PS-modified system has higher initial amplitude spikes and a more complex time evolution than pure proteinoids. This suggests that iron interacts better with the membrane in the hybrid structure.

The Fe^3+^ control mechanism boosts electrical signals in proteinoid systems. This boost connects to the shape changes seen in scanning electron microscopy (SEM), shown in Figure 2. Fe^3+^ ions bond with carboxylate groups (R-COO^–^) from aspartic and glutamic acids. This bonding boosts heterogeneous nucleation. As a result, it creates larger proteinoid microspheres, measuring 1.094 μm, compared to 0.277 μm in systems without doping. It also forms pyramidal PDLLA structures with base lengths of 21.6 μm. These shape changes happen because of a lower nucleation energy barrier (eq 3). This increases the surface area at the interface. As a result, it boosts ion binding and redox activity. The primary electrochemical driver is the reversible redox reaction

15 $${\mathrm{Fe}}^{3+}+e^{-}⇌{\mathrm{Fe}}^{2+}$$

This redox process boosts membrane potentials. Fe^3+^-doped proteinoid-PS systems show spikes up to 250 mV. In contrast, undoped systems only reach 5 to 30 mV (Figure 6). Larger microspheres and organized pyramidal structures lead to better charge transport efficiency. This is shown by the lower impedance (7.22 × 10^4^ Ω in PS hybrids compared to 2.03 × 10^5^ Ω in pure proteinoids, see Table 3) and a higher spike shape factor. The spike shape factor (SSF) is 0.15–0.20 with Fe^3+^ and 0.021–0.025 without it. These structural changes provide more stable and conductive pathways, enabling faster charge transfer kinetics (50% higher d*V*/d*t*). The signal sharpness (eq 14) also shows a big increase. This is calculated from the second derivative of the potential-time curve. Fe^3+^ ions change how charge moves at the membrane interface. This is especially true in the hybrid PS system, where a larger surface area helps more ions interact with the membrane. The combination of morphology and electricity highlights Fe^3+^ as a crucial modulator. It helps control action potential-like signals. This is important for biomolecular computing applications.

Frequency analysis (eq 9) shows a change. In pure systems, there are regular oscillations. However, in Fe^3+^-modified systems, the behavior becomes complex and multifrequent. This change is especially clear in the proteinoid-PS case. Initially, high-frequency spikes occur at about 0.5 Hz. Then, they gradually shift to lower-frequency oscillations around 0.1 Hz in the later phase.

A direct comparison of timing patterns in pure proteinoid and proteinoid-PS systems reveals different oscillation behaviors (see Figure 7). Pure proteinoids show random spiking with uneven amplitudes and intervals. In contrast, the proteinoid-PS system displays smooth sinusoidal oscillations described by

16 $$V\left(t\right)=A\sin\left(2\pi \mathrm{ft}\right)+V_{\mathrm{offset}}$$

where *A* = 1.5 mV represents the oscillation amplitude, *f* = 0.05 Hz is the characteristic frequency, and *V*_offset_ accounts for the baseline potential. This sinusoidal pattern has a peak-to-peak amplitude of 2–3 mV and occurs every 15–20 s. This suggests that PS incorporation changes how the membrane regulates charge transport. It shifts from random events to synchronized processes. Regular oscillations show better organization in the hybrid system’s electrochemical behavior. This may come from the ordered arrangement of proteinoid structures in the PS matrix. The difference matters. Pure proteinoids show irregular spiking between 4 and 10 mV. In contrast, the PS-modified system has a smooth sinusoidal pattern.

**Figure 7:**
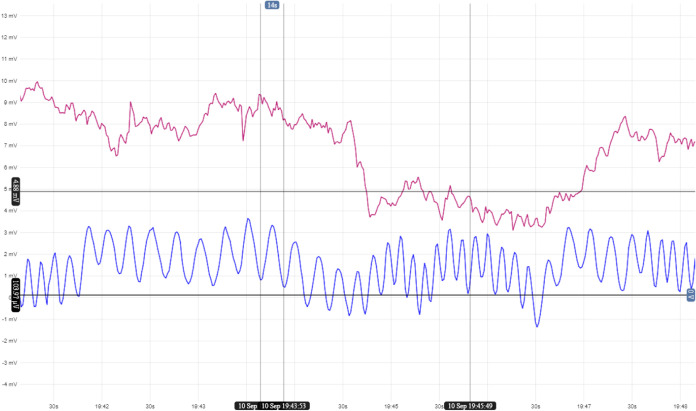
Comparing membrane potential changes in pure proteinoid (red line) and proteinoid-PS microspheres (blue line) during a 6 min recording. Pure proteinoids show irregular spikes. Their amplitudes range from 4 to 10 mV. The intervals are nonuniform, with sharp transitions and baseline drift. Proteinoid-PS systems show clear sinusoidal oscillations. They have a steady peak-to-peak amplitude of about 2–3 mV. These systems also exhibit a regular periodicity of around 15–20 s. The proteinoid-PS signal has a sinusoidal shape, represented by the equation *V*(*t*) = *A* sin­(2π*ft*) + *V*_offset_. Here, *A* is about 1.5 mV, and *f* is roughly 0.05 Hz. This pattern indicates a more structured charge transport process than the random spiking seen in pure proteinoids. The regular waveform in the PS-modified system shows coherent membrane potential oscillations. This may happen because of synchronized ion transport across the hybrid interface. The key difference in signal shape (SSF_PS_ ≈ 0.025 vs irregular SSF for pure proteinoids) shows that adding PS creates a more organized charge transport system.

### Electrochemical Characterization of Proteinoid Systems

We studied the electrochemical behavior of proteinoid assemblies using cyclic voltammetry (CV). We tested a potential range from −4 to +4 V. The fundamental electron transfer processes can be analyzed through several key electrochemical parameters and relationships.

#### Diffusion-Controlled Processes

The Randles-Sevcik equation was employed to analyze the peak current response

17 $$I_{p}=\left(2.69\times 10^{5}\right)n^{3/2}AD^{1/2}Cv^{1/2}$$

where *I*_p_ represents the peak current (A), *n* is the number of electrons transferred, *A* is the electrode surface area (cm^2^), *D* is the diffusion coefficient (cm^2^ s^–1^), *C* is the bulk concentration (mol cm^–3^), and *v* is the scan rate (V s^–1^).

#### Reversibility Analysis

The degree of electrochemical reversibility was evaluated through peak potential separation

18 $$\Delta E_{p}=\left|E_{\mathrm{pa}}-E_{\mathrm{pc}}\right|$$

For a reversible system, Δ*E*_p_ gets close to $\frac{59}{n}\;\mathrm{mV}$ . If the separations are larger, the processes are quasi-reversible or irreversible. The charge transfer coefficient (α) can be determined by

19 $$\alpha =\frac{47.7}{\Delta E_{p}}$$

#### Capacitive Behavior

The double-layer capacitance (*C*_dl_) was calculated using

20 $$C_{\mathrm{dl}}=\frac{i}{v}$$

where *i* represents the nonFaradaic current component. The total energy storage capacity was evaluated through hysteresis analysis

21 $$E_{\mathrm{stored}}=\oint IdV$$

#### Reaction Kinetics

The relationship between peak current and scan rate provides insight into the reaction mechanism

22 $$I_{p}\propto v^{1/2}\;\left(\mathrm{diffusion}‐\mathrm{controlled}\right)$$

23 $$I_{p}\propto v\;\left(\mathrm{surface}‐\mathrm{confined}\right)$$

The diffusion coefficient can be extracted from the slope of *I*_p_ vs *v* ^1/2^ plots using the rearranged Randles-Sevcik equation:

24 $$D={\left(\frac{I_{p}}{(2.69\times 10^{5})n^{3/2}\mathrm{AC}v^{1/2}}\right)}^{2}$$

In the electrochemical analysis of proteinoid systems, a needle-like cylindrical electrode was employed. The effective electrode area (*A*) is key for calculating current density. We determined it using the electrode’s geometric parameters. For a cylindrical electrode with diameter *d* = 0.1 mm, the radius (*r*) is given by

25 $$r=\frac{d}{2}=\frac{0.1\;\mathrm{mm}}{2}=0.05\;\mathrm{mm}=5\times 10^{-3}\;\mathrm{cm}$$

The cross-sectional area was then calculated using

26 $$A=\pi r^{2}=\pi {\left(5\times 10^{-3}\;\mathrm{cm}\right)}^{2}=7.85\times 10^{-5}\;{\mathrm{cm}}^{2}$$

We used this electrode area in the Randles–Sevcik equation (eq 17) to find the diffusion coefficient (*D*) of the electroactive species. The small electrode area (*A* = 7.85 × 10^–5^ cm^2^) keeps *iR* drop effects low. It also ensures a good signal-to-noise ratio for the Faradaic currents measured. The geometric surface area was key for normalizing the capacitive current contributions from eq 24. This made it possible to accurately determine the specific capacitance of the proteinoid-modified electrode surface.

We studied the electrochemical behavior of proteinoid structures using cyclic voltammetry for 100 cycles. Figure 8 shows the evolution of the current–voltage response across the potential window of −4 to +4 V. The voltammograms reveal a complex electrochemical profile with both Faradaic and capacitive contributions. The color gradient shifts from blue in early cycles to yellow in later cycles. This shows the changes in the electrochemical response. Peak currents vary from −3 to +2.5 mA.

**Figure 8:**
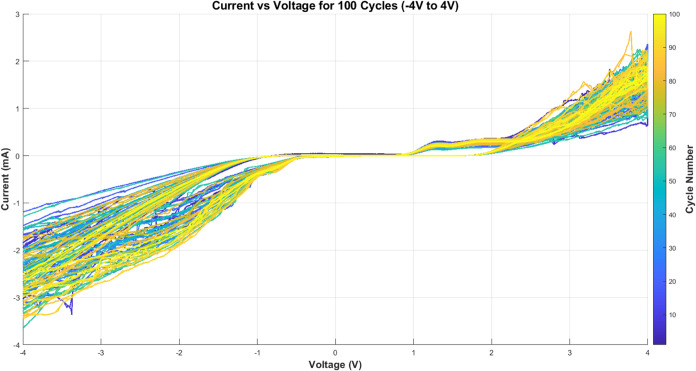
Cyclic voltammograms of proteinoids over 100 cycles between −4 and +4 V at a scan rate of 100 mV s^–1^. Color gradient indicates cycle progression (blue: early cycles, yellow: later cycles), demonstrating evolution of the electrochemical response. Peak currents range from −3 to +2.5 mA, with notable changes in the voltammetric profile across cycling.

A close look at the electrochemical parameters (Figure 9) shows how charge transfer works and highlights stability. The hysteresis area (Figure 9a) ranges from 1.37 × 10^–3^ to 3.79 × 10^–3^ J. This shows that the energy storage capacity changes during cycling. The peak potential separation Δ*E*_p_ (Figure 9b) stabilizes at about 7.9 V. This value is much higher than the theoretical 59 mV/*n* for reversible systems. It indicates a quasi-reversible electron transfer process.

**Figure 9:**
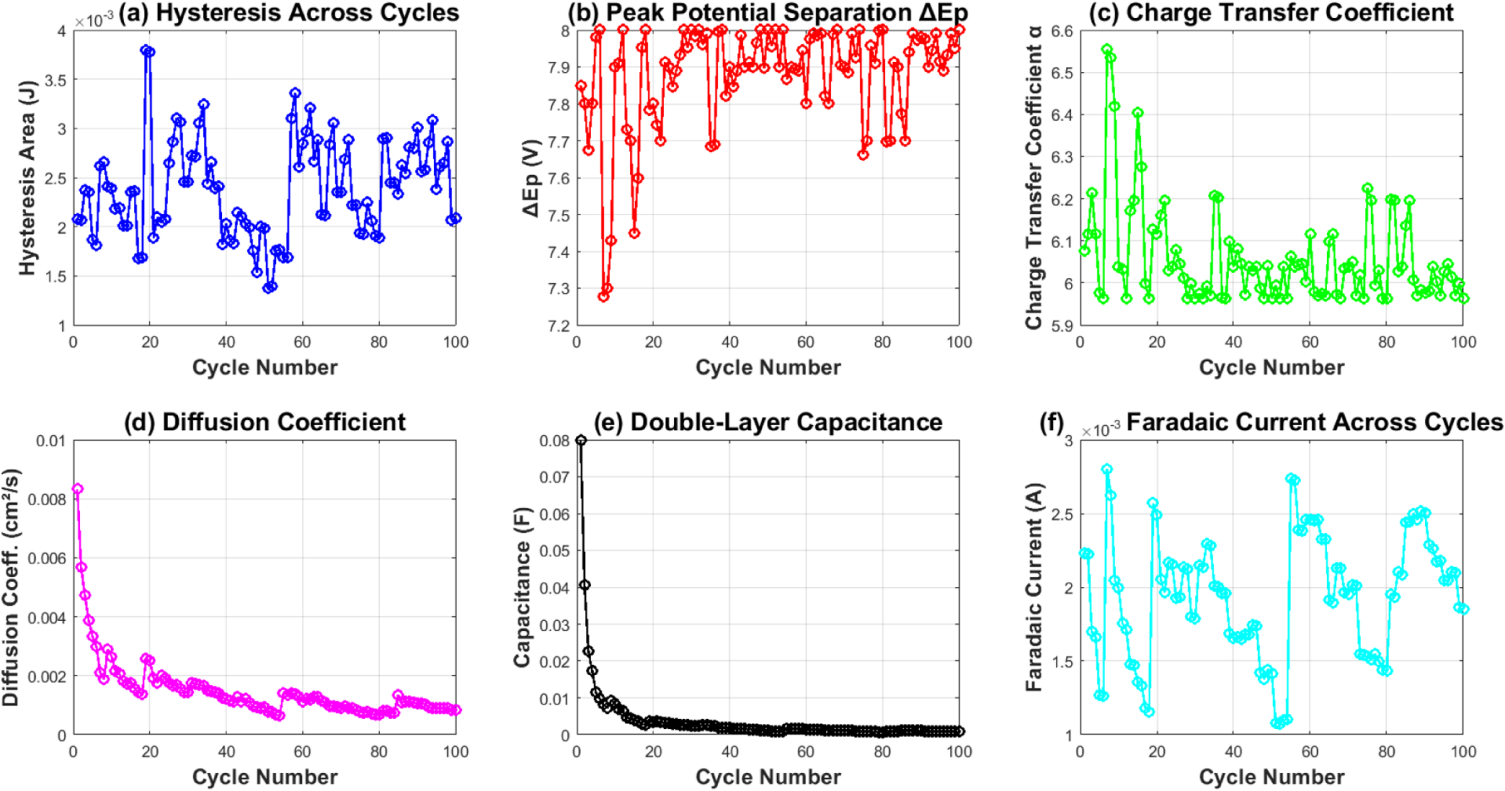
Evolution of key electrochemical parameters over 100 cycles: (a) Hysteresis area showing energy storage capacity fluctuating between 1.37 × 10^–3^ and 3.79 × 10^–3^ J; (b) peak potential separation (Δ*E*_p_) stabilizing around 7.9 V, indicating quasi-reversible electron transfer; (c) charge transfer coefficient (α) ranging from 5.96 to 6.55, reflecting electron transfer kinetics; (d) diffusion coefficient (*D*) decreasing from 8.33 × 10^–3^ to 8.30 × 10^–4^cm^2^ s^–1^; (e) double-layer capacitance (*C*_dl_) showing exponential decay from 7.99 × 10^–2^ to 7.81 × 10^–4^ F; (f) Faradaic current varying between 1.08 × 10^–3^ and 2.80 × 10^–3^ A, demonstrating sustained electrochemical activity.

The charge transfer coefficient, α (Figure 9c), ranges from 5.96 to 6.55. Meanwhile, the diffusion coefficient, *D* (Figure 9d), decreases from 8.33 × 10^–3^ to 8.30 × 10^–4^ cm^2^ s^–1^. This shows that mass transport is gradually getting restricted. The double-layer capacitance *C*_dl_ (Figure 9e) shows an exponential drop from 7.99 × 10^–2^ to 7.81 × 10^–4^ F. This indicates that there is structural change at the electrode interface. Even with these changes, the Faradaic current (Figure 9f) stays active. It ranges from 1.08 × 10^–3^ to 2.80 × 10^–3^ A during cycling. This shows that the proteinoid system keeps its electrochemical activity.

Table 1 shows key data on the stability and performance of the proteinoid structures. The charge transfer coefficient (α) has a stable mean value of 5.47 ± 0.16. This shows that electron transfer kinetics remain consistent during cycling. Similarly, the peak separation shows minimal variation (8.73 ± 0.24 V), further supporting stable electrode kinetics.

**Table 1:** Statistical Analysis of Key Electrochemical Parameters for Proteinoid Structures over 100 Cycles^a^

parameter	mean	Std Dev	min	max
hysteresis area (J)	2.54 × 10^–3^	4.80 × 10^–4^	1.58 × 10^–3^	3.69 × 10^–3^
peak separation (V)	8.73	2.38 × 10^–1^	7.90	9.00
α (dimensionless)	5.47	1.55 × 10^–1^	5.30	6.04
diffusion Coeff. (cm^2^ s^–1^)	2.07 × 10^–3^	1.41 × 10^–3^	8.75 × 10^–4^	1.08 × 10^–2^
capacitance (F)	4.35 × 10^–3^	1.04 × 10^–2^	8.12 × 10^–4^	9.08 × 10^–2^
Faradaic current (A)	1.86 × 10^–3^	4.84 × 10^–4^	1.04 × 10^–3^	4.02 × 10^–3^
peak current (A)	2.43 × 10^–3^	6.12 × 10^–4^	1.44 × 10^–3^	4.86 × 10^–3^

a The hysteresis area (J), peak separation (V), charge transfer coefficient (*α*), diffusion coefficient (cm^2^ s^–1^), double-layer capacitance (F), Faradaic current (A), and peak current (A) show the range of electrochemical behavior. The low standard deviations in α and peak separation show stable electron transfer kinetics. Larger changes in the diffusion coefficient and capacitance show that mass transport and interfacial properties are changing.

The diffusion coefficient varies widely. It ranges from 8.75 × 10^–4^ to 1.08 × 10^–2^ cm^2^ s^–1^. The average is 2.07 × 10^–3^ cm^2^ s^–1^. This substantial range (σ = 1.41 × 10^–3^ cm^2^ s^–1^) suggests dynamic changes in mass transport properties during cycling. The double-layer capacitance varies a lot, ranging from 8.12 × 10^–4^ to 9.08 × 10^–2^ F. This change shows how interfacial properties evolve.

The Faradaic and peak currents stay within consistent ranges. Their mean values are 1.86 × 10^–3^ and 2.43 × 10^–3^ A. This shows that electrochemical activity remains steady. The hysteresis area shows how much energy can be stored. It varies moderately, with a mean of 2.54 × 10^–3^ J and a standard deviation of 4.80 × 10^–4^ J. This indicates good stability in the electrochemical performance.

### Electrochemical Characterization of Proteinoid–PS Systems

The electrochemical behavior of proteinoid-polystyrene hybrids is different from pure proteinoid systems. Figure 10 shows cyclic voltammograms over 100 cycles. The current response varies from −3 to +5 mA. This is much higher than that of the pure proteinoid system. The color change from blue to yellow shows how the electrochemical response evolves. This suggests that the interface changes during cycling.

**Figure 10:**
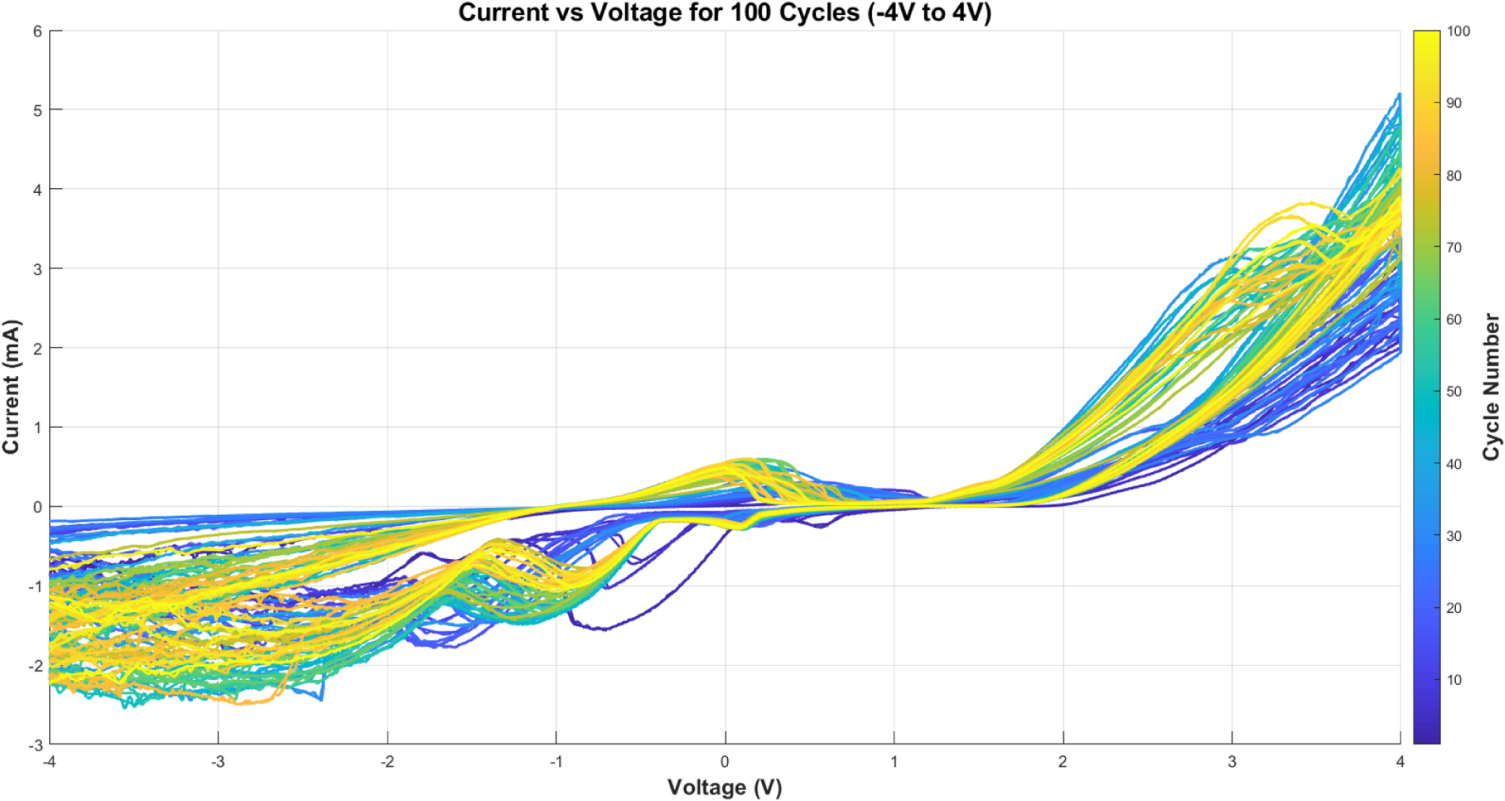
Cyclic voltammograms of proteinoid-polystyrene microsphere composites over 100 cycles between −4 and +4 V at 100 mV s^–1^. The color gradient shows cycle progression: blue is for early cycles, and yellow is for later cycles. This reveals a better current response, changing from −3 to +5 mA. This improvement, compared to pure proteinoids, suggests enhanced charge transfer in the hybrid system.

The parameter analysis in Figure 11 shows important differences in electrochemical properties. The hysteresis area (Figure 11a) increases significantly. It reaches 6.5 × 10^–3^ J. This shows a better energy storage capacity than pure proteinoids. The statistical analysis in Table 2 backs this up. It shows that the hybrid system has a mean hysteresis area of 4.20 × 10^–3^ J. In contrast, pure proteinoids have a mean of 2.54 × 10^–3^ J (Table 1).

**Figure 11:**
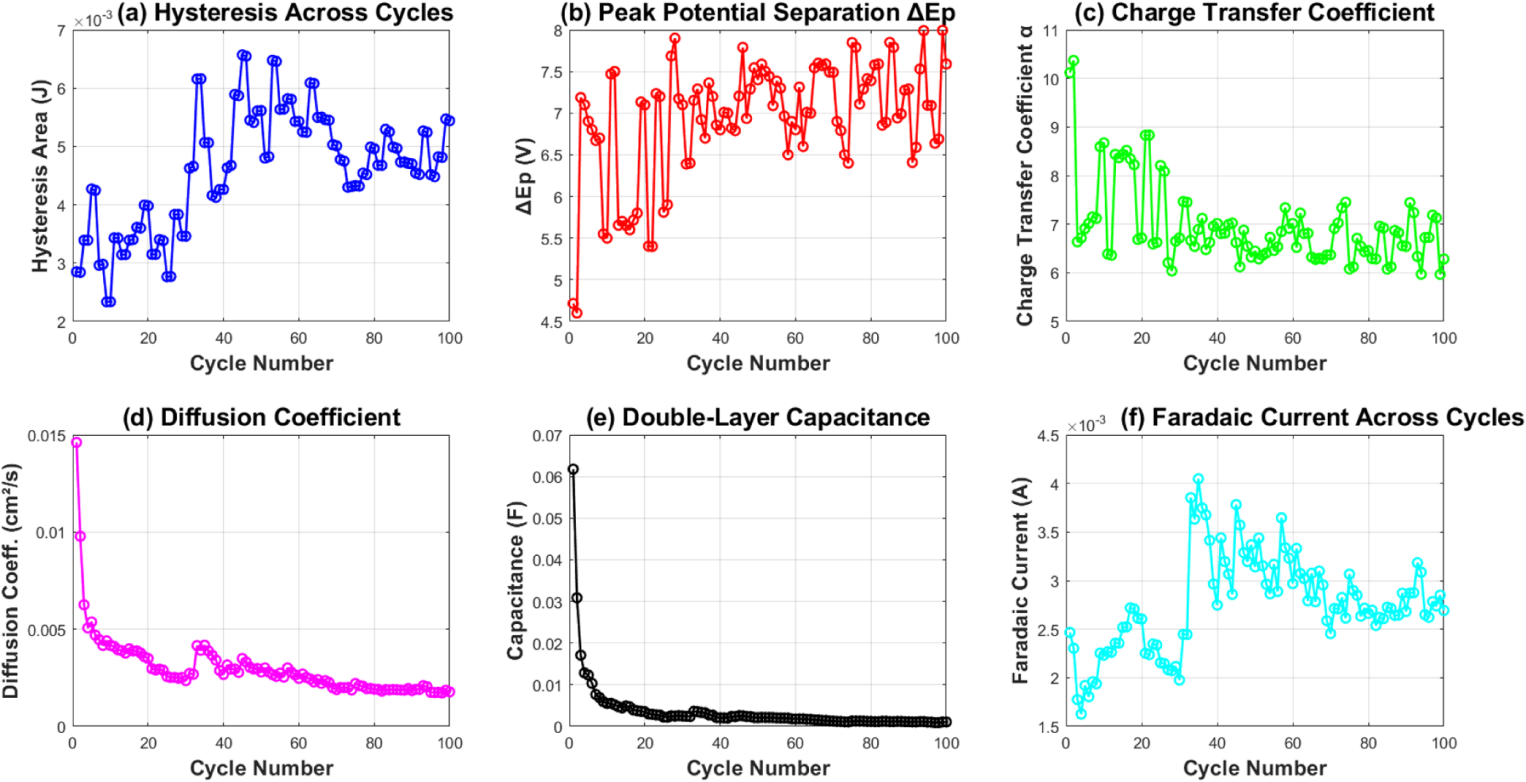
Electrochemical variations in proteinoid-polystyrene composites over 100 cycles: (a) Hysteresis area shows energy storage rising from 2.5 to 6.5 × 10^–3^ J; (b) peak potential separation (Δ*E*_p_) stabilizes at 7.5 V after some initial changes, showing quasi-reversible electron transfer; (c) charge transfer coefficient increases to α = 6 to 10; (d) diffusion coefficient (*D*) drops from 1.5 × 10^–2^ to 2 × 10^–3^ cm^2^ s^–1^; (e) double-layer capacitance (*C*_dl_) decreases from 6 × 10^–2^ to 3 × 10^–3^ F; (f) Faradaic current remains steady between 1.5 × 10^–3^ and 4.0 × 10^–3^ A. The composite system demonstrates enhanced electrochemical performance compared to pure proteinoids.

**Table 2:** Statistical Analysis of Key Electrochemical Parameters for Proteinoid-Polystyrene Hybrid Structures over 100 Cycles^a^

parameter	mean	Std Dev	min	max
hysteresis area (J)	4.20 × 10^–3^	1.09 × 10^–3^	1.86 × 10^–3^	6.38 × 10^–3^
peak separation (V)	7.92	7.29 × 10^–1^	5.68	9.00
α (dimensionless)	6.08	6.43 × 10^–1^	5.30	8.39
diffusion Coeff. (cm^2^ s^–1^)	4.83 × 10^–3^	2.99 × 10^–3^	2.84 × 10^–3^	2.68 × 10^–2^
capacitance (F)	5.48 × 10^–3^	1.12 × 10^–2^	1.32 × 10^–3^	9.97 × 10^–2^
Faradaic current (A)	4.64 × 10^–3^	1.21 × 10^–3^	2.41 × 10^–3^	7.15 × 10^–3^
peak current (A)	5.96 × 10^–3^	1.50 × 10^–3^	3.21 × 10^–3^	8.97 × 10^–3^

a The enhanced hysteresis area (J), modified peak separation (V), charge transfer coefficient (α), diffusion coefficient (cm^2^ s^–1^), double-layer capacitance (F), Faradaic current (A), and peak current (A) demonstrate altered electrochemical behavior compared to pure proteinoids. The increased mean values and standard deviations suggest more dynamic electrochemical processes in the hybrid system.

The charge transfer kinetics improved significantly. The charge transfer coefficient, α, rose from an average of 5.47 in pure proteinoids to 6.08 in the hybrid system. It peaked at 8.39 (see Figure 11c). The diffusion coefficient shows better mass transport. Its average value is 4.83 × 10^–3^ cm^2^ s^–1^. In pure proteinoids, the value is 2.07 × 10^–3^ cm^2^ s^–1^.

The increase in Faradaic current is especially important. The hybrid system shows a mean of 4.64 × 10^–3^ A, while pure proteinoids show 1.86 × 10^–3^ A. This means the hybrid system has more efficient electron transfer processes. The double-layer capacitance stays within similar ranges. However, it behaves more steadily in the hybrid system. This indicates improved interfacial stability.

Adding polystyrene to the proteinoid structure boosts electrochemical performance. This change leads to higher current responses, faster charge transfer, and improved energy storage. These improvements come from the composite structure working together. Polystyrene likely adds extra conductive pathways and helps stabilize the proteinoid system.

### Impedance-Based Equivalent Circuit Analysis: Comparing Pure Proteinoids and Polystyrene-Modified Systems

We performed a detailed analysis of the electrochemical impedance of proteinoid systems using different methods. The Nyquist plots in Figure 12 show clear differences between pure proteinoids and PS-modified systems. The pure proteinoid mainly shows capacitive behavior. Its *Z*″ value reaches 3000 kΩ. In contrast, the PS composite features a semicircle, which suggests better charge transfer processes.

**Figure 12:**
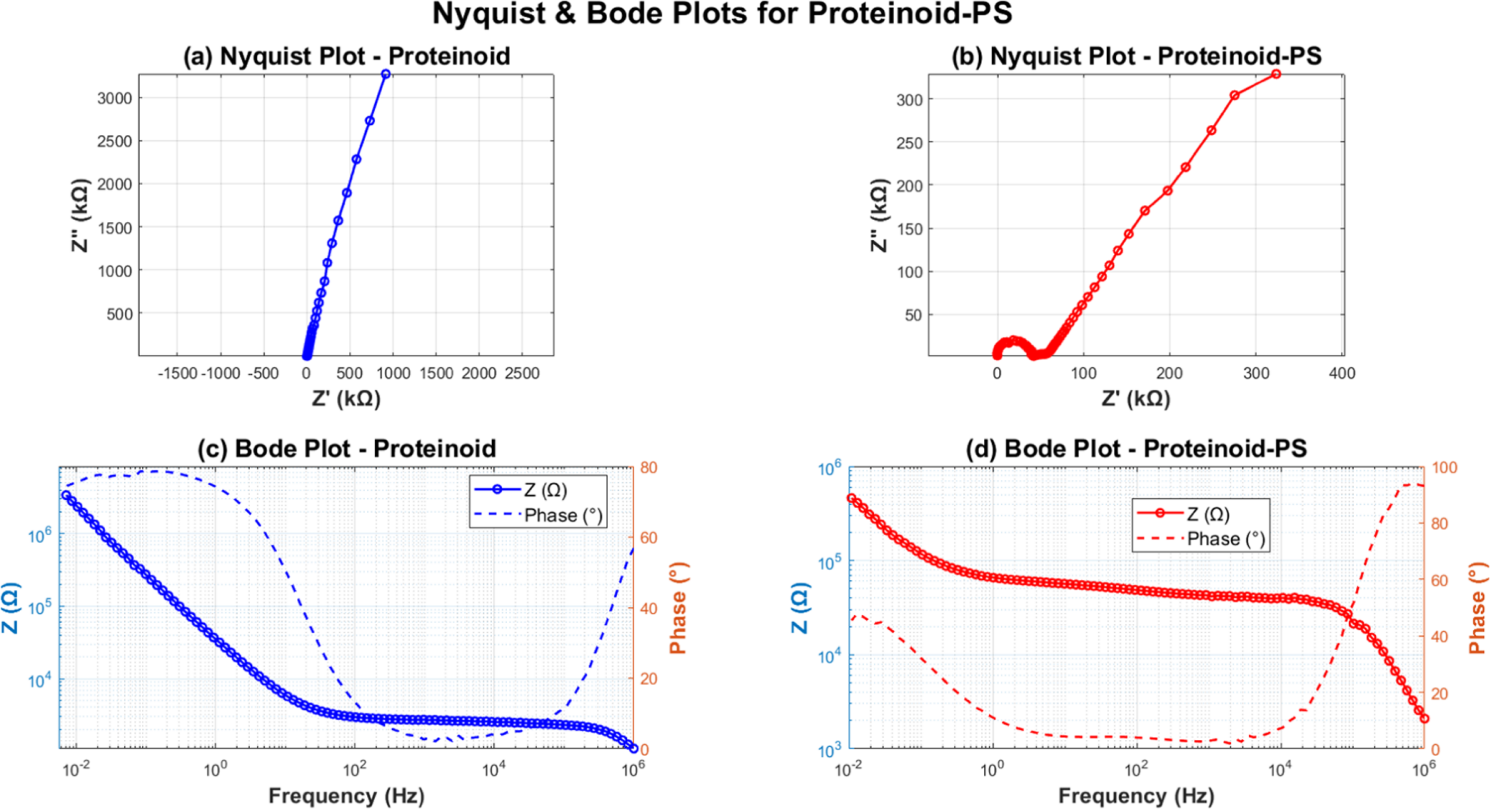
Electrochemical impedance spectroscopy compared pure proteinoids and proteinoid-polystyrene (PS) composites: (a) The Nyquist plot for pure proteinoids shows a near-linear response. Here, *Z*″ reaches 3000 kΩ, indicating mainly capacitive behavior. (b) The Nyquist plot for the proteinoid-PS composite features a semicircle at high frequencies (*Z*″ ≈ 300 kΩ). This suggests better charge transfer processes due to a diffusion-controlled region. (c) The Bode plot for pure proteinoids shows impedance magnitude (|*Z*|) decreasing from 10^6^ to 10^3^ Ω as frequency increases. Phase angle shifts indicate a transition from capacitive to resistive behavior. (d) The Bode plot for the proteinoid-PS composite displays overall impedance reduced from 10^5^ to 10^3^ Ω. The phase angle variations suggest a more complex interfacial process, with multiple time constants. The frequency range spans from 10^–2^ to 10^5^ Hz for all measurements.

The Bode plots highlight these differences. The impedance magnitude of pure proteinoids ranges from 10^6^ to 10^3^ Ω. In contrast, the PS composite has a smaller range of 10^5^ to 10^3^ Ω (Figure 12c,d). The impedance reduction is shown in Table 3. The mean |*Z*| drops from 2.03 × 10^5^ Ω for pure proteinoids to 7.22 × 10^4^ Ω for the PS composite.

**Table 3:** Comparative Analysis of Electrochemical Impedance Parameters for Pure Proteinoids and Proteinoid-Polystyrene (PS) Composites^a^

parameter	mean	Std Dev	min	max
*Z*′prot (kΩ)	49.48	144.09	0.60	914.25
*Z*′PS (kΩ)	61.29	55.74	–0.31	323.58
*Z*″prot (kΩ)	195.75	557.67	0.09	3273.49
*Z*″PS (kΩ)	30.22	62.87	1.38	329.06
|*Z*|prot (Ω)	2.03 × 10^5^	5.76 × 10^5^	1.09 × 10^3^	3.40 × 10^6^
|*Z*|PS (Ω)	7.22 × 10^4^	8.07 × 10^4^	2.09 × 10^3^	4.62 × 10^5^
ϕ_prot_ (°)	38.11	30.93	2.01	78.75
ϕ_PS_ (°)	23.51	26.80	1.88	93.98

a The real impedance (*Z*′), imaginary impedance (*Z*″), magnitude impedance (|*Z*|), and phase angle (ϕ) show different interfacial features between the systems. The lower impedance values and phase angles in the PS composite show better charge transfer. This means less resistance at the electrode interface.

Equivalent circuit modeling (see Figure 13) shows the mechanisms that cause these differences. The pure proteinoid system is well-described by a simple *R*(*WC*) circuit with *R*_*s*_ = 2.106 kΩ and *C* = 4.229 μF (Table 4). The PS composite needs a more complex (*WC*) *O* circuit. It includes a finite-length diffusion element (*O*) with σ = 2.386 × 10^7^ and a time constant of τ = 2.000 × 10^–3^ s.

**Figure 13:**
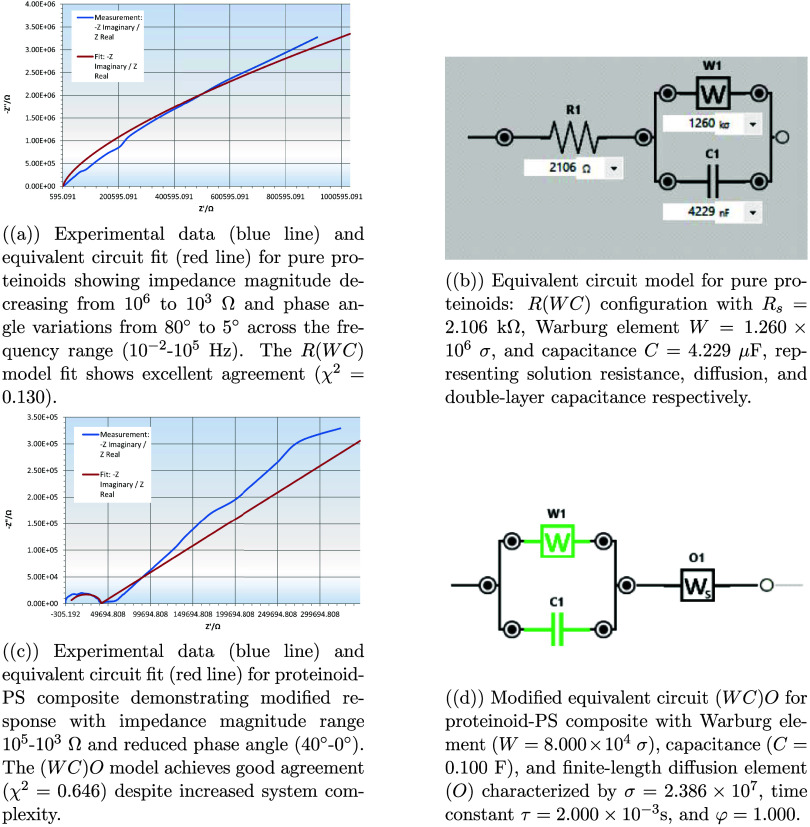
Equivalent circuit analysis comparing pure proteinoid and proteinoid-PS systems. The transition from *R*(*WC*) to (*WC*) *O* circuit models reflects the introduction of finite-length diffusion processes in the hybrid system, while maintaining accurate impedance response fitting across the full frequency range (10^–2^–10^5^ Hz).

**Table 4:** Parameters from Electrochemical Impedance Spectroscopy for Pure Proteinoid and Proteinoid-Polystyrene (PS) Systems are Shown in the Equivalent Circuit Fitting^a^

system	element	fitted value	unit	error (%)	χ^2^
proteinoid	*R*_1_	2.106 × 10^3^	Ω	5.17	0.130
	*W*_1_	1.260 × 10^6^	σ	12.51	
	*C*_1_	4.229 × 10^–6^	F	8.01	
proteinoid-PS	*W*_1_	8.000 × 10^4^	σ	15.10	0.646
	*C*_1_	1.000 × 10^–1^	F	9.25	
	*O*_1*a*_	2.386 × 10^7^	σ	15.42	
	*O*_1*b*_	2.000 × 10^–3^	√s	26.61	
	*O*_1*c*_	1.000	φ	36.92	

a The circuit elements are Solution resistance (*R*_s_), Warburg impedance (*W*), capacitance (*C*), constant phase element parameters (σ, φ). The goodness of fit is indicated by χ^2^ values and iteration counts. The proteinoid-PS system needed a more complex circuit model, (*WC*) *O*. This is different from the simpler *R*(*WC*) model for pure proteinoids. The added complexity shows more interfacial processes in the hybrid structure. Lower error percentages and χ^2^ values indicate reliable fitting results for both systems.

Both systems have excellent fitting quality. The χ^2^ values are 0.130 for pure proteinoids and 0.646 for PS-modified proteinoids. This is notable given the added complexity of the latter. The phase angle behavior highlights this clearly. The PS composite displays multiple time constants (Figure 13c). In contrast, pure proteinoids show a simpler response. The lower phase angles in the PS system (mean ϕ = 23.51° compared to 38.11°) support the improved charge transfer features of the hybrid interface.

The impedance characterization reveals significant differences between pure proteinoid and hybrid systems. As shown in Figure S2, adding PS cuts the mean impedance by about 3 times. It drops from |**Z**| ≈ 0.20 MΩ in pure proteinoids to |**Z**| ≈ 0.07 MΩ in proteinoid-PS hybrids. This means better electrical conductivity and lower interfacial resistance. Also, the impedance distribution analysis in Figure S3 shows that pure proteinoid systems have very variable electrical properties. Some outliers reach up to 3.4 MΩ. In contrast, proteinoid-PS hybrids cluster closely around 0.1 MΩ with little variation. This big jump in conductivity and reproducibility shows that PS templating makes standard bioelectronic interfaces. These are key for dependable biomolecular computing uses.

The resistance of the Fe­(NO_3_)_3_ solution was measured for 60,000 s (Figure S1, Supporting Information). It showed stable electrical behavior. The resistance stayed between 0.13 and 0.29 MΩ during the entire period. The smoothed resistance data show little change around a mean of about 0.15 MΩ. The trend line has a slight slope of approximately −0.0018 MΩ/s. This means that any small variations are likely due to experimental noise, rather than real electrical changes. This baseline stability of the Fe^3+^ solution is particularly significant, as it establishes that Fe^3+^ ions in aqueous solution do not exhibit spontaneous resistance variations, electrical oscillations, or time-dependent conductivity changes. The steady electrical properties of the ferric nitrate solution show that Fe^3+^ ions maintain a stable redox balance in solution, even without any external influence during the measurement period. These findings provide important baseline data that strongly contrast with the electrical behaviors observed in Fe^3+^-doped proteinoid systems. In such systems, dramatic resistance fluctuations, oscillatory patterns, and large-amplitude modulations are seen. This confirms that the observed bioelectronic phenomena require the specific proteinoid microenvironment and cannot be explained by the properties of the Fe^3+^ solution alone.

We studied the electrical behavior of Fe­(NO_3_)_3_ solution. This helped us find baseline properties. It also confirmed that the dynamic bioelectronic phenomena seen in proteinoid systems are not just due to Fe^3+^ ions. The Fe­(NO_3_)_3_ solution shows a steady potential profile over 12,000 s, as seen in Figure S4. It starts with a quick drop from +120 mV to about −50 mV in the first 1500 s. Then, it stabilizes around −5 mV for the rest of the time. No spontaneous electrical oscillations, periodic spikes, or rhythmic variations appeared during the measurements. This confirms that there was no bioelectronic activity in the Fe^3+^ solution. This baseline behavior is very different from the complex oscillatory dynamics seen in proteinoid-Fe^3+^ hybrid systems. These systems show membrane potential spikes of up to 250 mV and perform computational logic operations. The smooth recovery curve shows that the signals and electrical patterns in our bioelectronic systems come from proteinoid-mediated redox processes. They do not arise from the natural traits of Fe^3+^ ions in water. This confirms that the interactions between proteinoids and Fe^3+^ are key to our magnetic control abilities.

### Implementation of Boolean Logic Operations in Pure and PS-Modified Proteinoid Microspheres

The implementation of Boolean logic operations using proteinoid systems can be formally defined through the following equations. The AND gate operation follows

27 $$\mathrm{AND}\left(A,B\right)=\{\begin{array}{l} 1,,\mathrm{if}\;V_{A}>V_{\mathrm{th}}\;\mathrm{AND}\;V_{B}>V_{\mathrm{th}}\\ 0,,\mathrm{otherwise} \end{array}$$

The OR gate is characterized by

28 $$\mathrm{OR}\left(A,B\right)=\{\begin{array}{l} 1,,\mathrm{if}\;V_{A}>V_{\mathrm{th}}\;\mathrm{OR}\;V_{B}>V_{\mathrm{th}}\\ 0,,\mathrm{otherwise} \end{array}$$

The XOR operation implements

29 $$\mathrm{XOR}\left(A,B\right)=\{\begin{array}{l} 1,,\mathrm{if}\;\left(V_{A}>V_{\mathrm{th}}\;\mathrm{XOR}\;V_{B}>V_{\mathrm{th}}\right)\\ 0,,\mathrm{otherwise} \end{array}$$

Finally, the NOT gate performs signal inversion according to

30 $$\mathrm{NOT}\left(A\right)=\{\begin{array}{l} 1,,\mathrm{if}\;V_{A}<V_{\mathrm{th}}\\ 0,,\mathrm{if}\;V_{A}<V_{\mathrm{th}} \end{array}$$

In these equations, *V*_th_ = 5 mV represents the threshold voltage that determines the binary state transition. Figure 14 shows how pure proteinoid and proteinoid-PS systems perform basic logic operations. The PS modification mainly impacts how the NOT gate switches.

**Figure 14:**
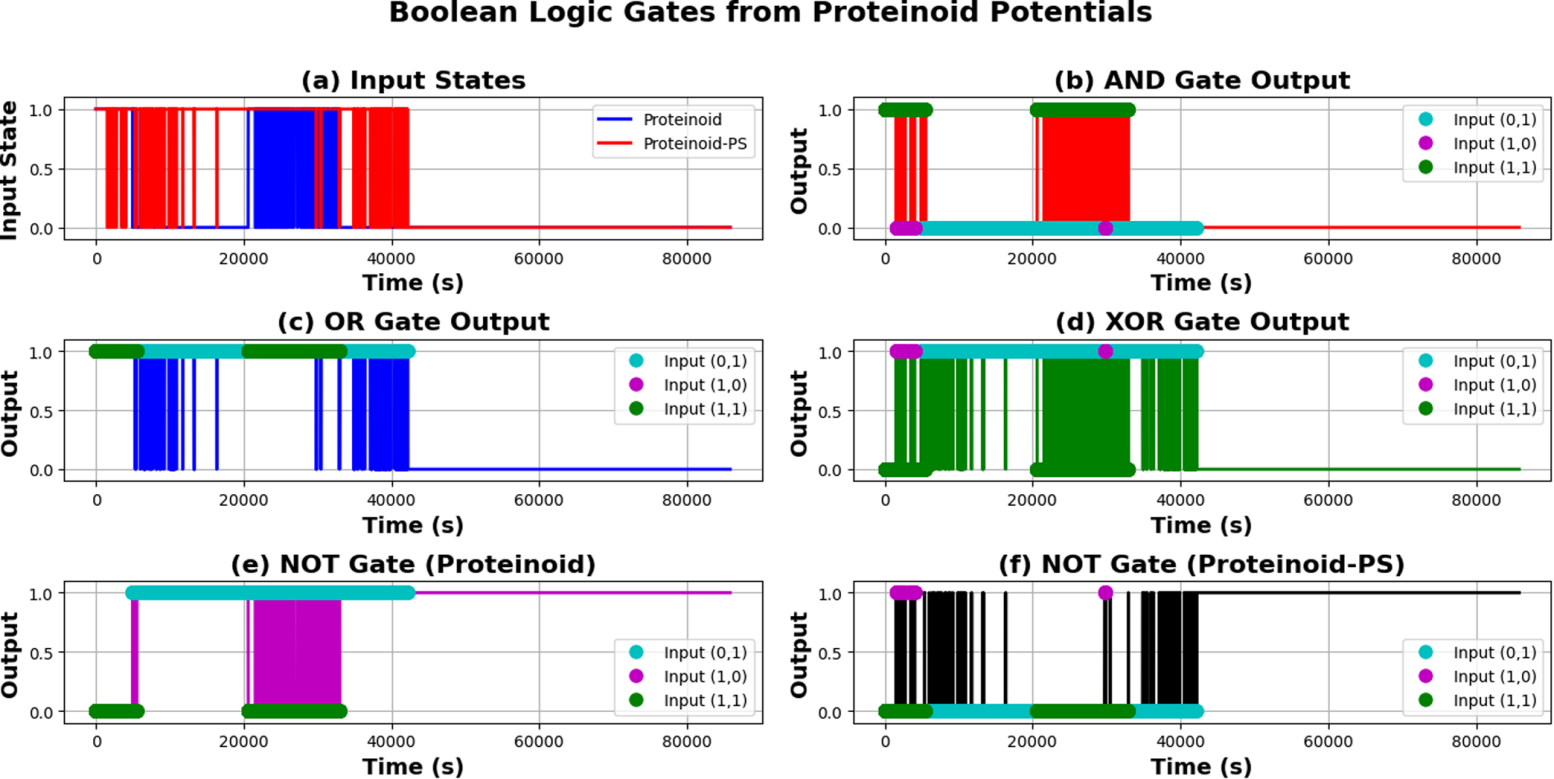
Binary logic operations implemented using proteinoid and proteinoid-PS membrane potentials with threshold voltage (*V*_th_) of 5 mV. (a) Input states: The proteinoid (blue) and proteinoid-PS (red) potentials are thresholded to binary states (0 or 1), showing frequent switching for proteinoid early on (0 to 20,000 s) and stabilizing at 1 (20,000 to 60,000 s), while proteinoid-PS remains mostly 0 with brief spikes at 20,000, 40,000, and 60,000 s. (b) AND gate output: Outputs high (1, red line) only when both inputs are 1 (green dots for (1,1)), staying low for (0,1) (cyan dots) and (1,0) (magenta dots), with high states around 20,000, 40,000, and 60,000 s. (c) OR gate output: Outputs high (1, blue line) when at least one input is 1 (cyan, magenta, green dots), remaining high most of the time due to the proteinoid input, dropping to 0 only when both inputs are 0. (d) XOR gate output: Outputs high (1, green line) when inputs differ (cyan for (0,1), magenta for (1,0)), such as between 20,000 and 40,000 s, and low for (1,1) (green dots). (e) NOT gate (proteinoid): Inverts the proteinoid input (magenta line), high when proteinoid is 0 (e.g., 0 to 20,000 s, after 60,000 s), with input combinations marked (cyan, magenta, green dots). (f) NOT gate (proteinoid-PS): Inverts the proteinoid-PS input (black line), mostly high due to proteinoid-PS being 0, with brief drops at 20,000, 40,000, and 60,000 s (cyan, green dots). All operations maintain stable performance for more than 80,000 s. Proteinoid-PS systems demonstrate improved switching dynamics, with sharper transitions in the NOT gate.

Fe­(NO_3_)_3_ affects how proteinoid systems spike. This shows key changes in membrane electrochemistry. When Fe^3+^ ions come into the system, they attach to the negatively charged carboxylate groups (COO^–^) from aspartic and glutamic acid on the proteinoid surface. This interaction establishes new charge distribution patterns across the membrane interface. The primary mechanism involves redox-mediated charge transport

31 $${\mathrm{Fe}}^{3+}+e^{-}⇌{\mathrm{Fe}}^{2+}$$

This reversible electron transfer helps separate charges across the membrane. This leads to greater potential differences. The coordination between Fe^3+^ and carboxylate groups can be expressed as

32 $$R‐{\mathrm{COO}}^{-}+{\mathrm{Fe}}^{3+}⇌R‐\mathrm{COO}\text{−}{\mathrm{Fe}}^{2+}$$

The experimental results (Figure 5) show stronger spiking behavior. Potentials hit 250 mV, much higher than the 5–30 mV range seen in undoped systems. The timing of these spikes shows a complex mix of ion binding, charge shifts, and changes to the membrane structure. In proteinoid-PS hybrid systems (Figure 6), the effect of Fe^3+^ stands out more. This happens because the interfacial area increases and the charge transport pathways change. PS adds more coordination sites and changes the local electric field distribution. This results in distinct temporal patterns characterized by

33 $$V\left(t\right)=V_{0}e^{-t/\tau }+A\sum_{i}\sin\left(\omega_{i}t+\phi_{i}\right)$$

where τ is the decay time and ω_*i*_ represents the oscillation modes from the Fe^3+^ processes. Fe^3+^ doping changes the membrane’s electrical behavior. It affects charge distribution, transport mechanisms, and the structure at the molecular level. This demonstrates a fundamental alteration in the electrochemical properties.

The changes in logic gate outputs are important for unconventional computing uses. Proteinoid systems show dynamic logic behavior. Their outputs change over time, unlike traditional semiconductor gates that stay the same.^70^

Proteinoid-based logic systems show unique behavior, as seen in Figure 15. The correlation analysis (Figure 15a) shows a moderate link between proteinoid and PS dynamics. The correlation coefficient is ρ = 0.4173. This partial correlation shows that the systems share some timing features. However, they have different computational properties.

**Figure 15:**
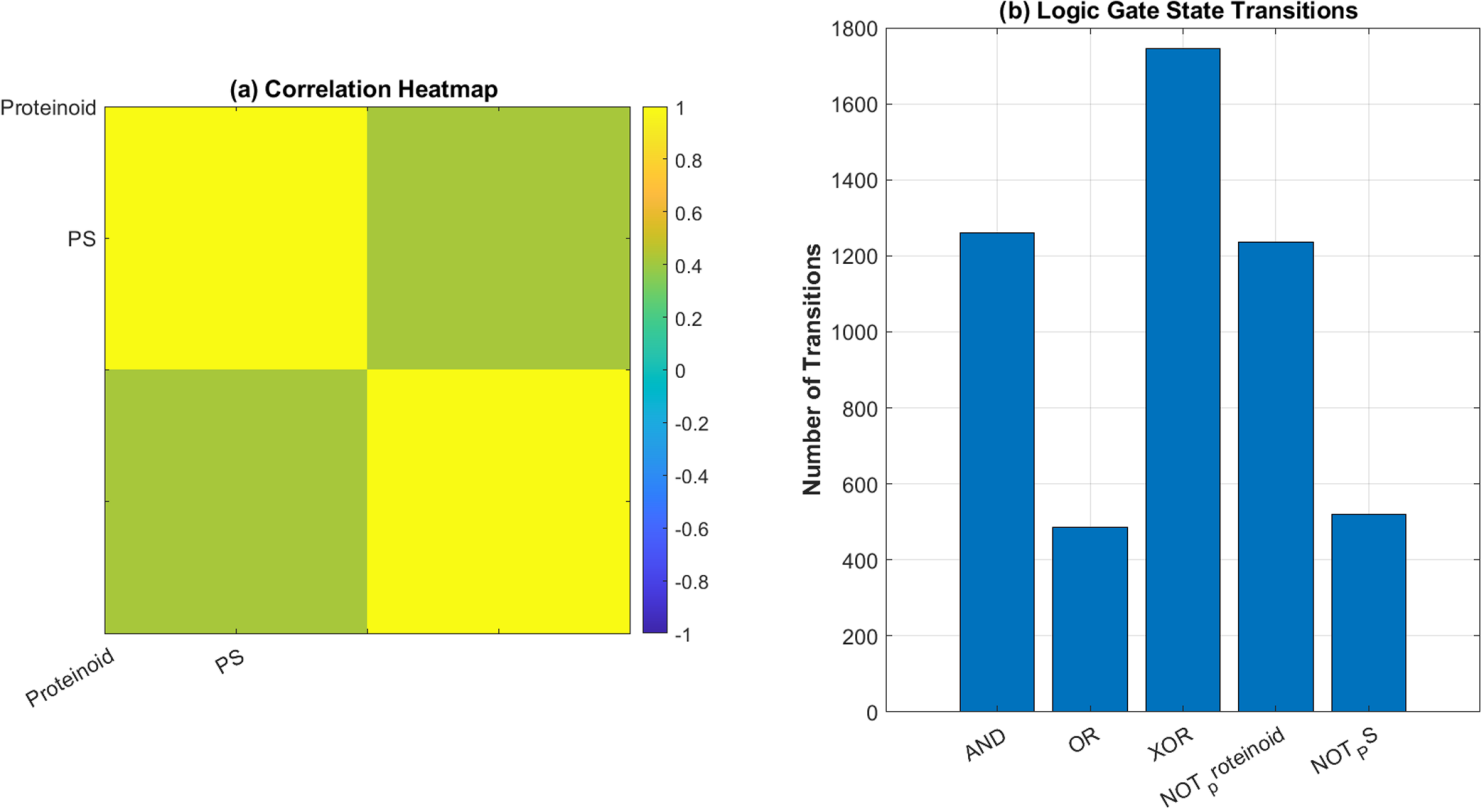
Analysis of logic gate dynamics in proteinoid and proteinoid-PS systems. (a) The correlation heatmap shows a moderate link (ρ = 0.4173) between proteinoid and PS signals. Yellow areas indicate positive self-correlation, while green areas show cross-correlation. (b) We measured state transitions for various logic operations. XOR had the most transitions, with 1744. Next was AND with 1261, and NOT proteinoid had 1235. The OR and NOT PS operations show reduced transition counts (485 and 519 respectively), suggesting more stable states.

The transition analysis (Figure 15b) measures how different logic operations switch dynamically. The XOR gate shows high activity with 1744 transitions. This indicates it is sensitive to state differences between the proteinoid and PS signals. The AND gate and NOT proteinoid operations have similar transition frequencies: 1261 and 1235. This suggests they have comparable dynamic complexity. The OR and NOT PS gates show fewer transitions, with 485 and 519 respectively. This means they have more stable operational states and longer persistence times.

The state transitions are defined by a threshold voltage (*V*_th_)­

34 $$S\left(t\right)=\{\begin{array}{l} 1,,\mathrm{if}\;V\left(t\right)>V_{\mathrm{th}}=5\;\mathrm{mV}\\ 0,,\mathrm{otherwise} \end{array}$$

The transition count (*T*_c_) for each logic gate is calculated as

35 $$T_{c}=\sum_{i=1}^{n-1}\left|S\left(i+1\right)-S\left(i\right)\right|$$

The stability analysis shows the percentage of time each gate maintains a stable state

36 $$\mathrm{stability}\;\left(\%\right)=\frac{\sum_{i=1}^{n}\left(S_{i}=\mathrm{mode}\left(S\right)\right)}{n}\times 100$$

The correlation coefficient (ρ) between proteinoid and PS potentials

37 $$\rho =\frac{\mathrm{cov}\left(V_{\mathrm{prot}},V_{\mathrm{PS}}\right)}{\sigma_{V_{\mathrm{prot}}}\sigma_{V_{\mathrm{PS}}}}=0.4173$$

The entropy (*H*) of each logic gate, measuring information content

38 $$H=-p\log_{2}\left(p\right)-\left(1-p\right)\log_{2}\left(1-p\right)$$

where *p* is the probability of the high state.

The behavioral patterns, along with the entropy measurements (*H*_OR_ = 0.9938, *H*_XOR_ = 0.8921) and stability analysis (AND stability = 85.53%), show a complex computational landscape. Here, different logic operations have unique time traits and information processing abilities.

The most interesting aspect is how different gates show distinct temporal patterns:

- The AND gate shows clear periods of activity followed by sustained low states.
- The OR gate maintains high states with intermittent drops.
- The XOR gate exhibits more complex switching behavior.
- The NOT gates display distinct patterns between pure proteinoid and proteinoid-PS systems.

This time-based behavior can help with temporal logic operations. These operations rely on both current inputs and their history. Potential applications include:

1. Pattern recognition systems that respond to temporal sequences.
2. Adaptive computing elements that can modify their response based on prior states.
3. Bioinspired information processing that mimics the dynamic nature of neural computation.
4. Signal processing applications where temporal filtering is desired.

The unique behavior of proteinoid-PS systems differs from pure proteinoids. We can change the material composition to adjust the computational properties. This shows a way to make biomolecular computing elements with specific timing features.

This unconventional computing goes beyond the usual binary logic gates. It opens up new ways for systems to compute by using dynamic state changes instead of fixed logical operations.

## Conclusions

We explored proteinoid and proteinoid-PS microsphere systems. They show unique electrochemical behavior and hold promise for computation. The electrical signals show a clear difference. In pure proteinoids, there is stochastic spiking. In PS hybrids, the signals have ordered sinusoidal oscillations at *f* = 0.05 Hz. This highlights how changes in structure can have a substantial impact on charge transport dynamics. Using Boolean logic with time-based changes opens doors for new computing methods. In this approach, how states change over time plays a key role in processing information. Fe^3+^ doping causes a strong reaction. This reaction involves the redox balance Fe^3+^ + e^–^ ⇌ Fe^2+^. It also leads to increased potential differences, around Δ*V* ≈ 250 mV. This allows for external control of these systems. The equivalent circuit analysis shifts from *R*(*WC*) to (*WC*) *O* topology. This transition helps us see more complex interfacial processes in pure and PS-modified systems. These findings help us understand primitive bioelectrical systems better. They also show that proteinoid microspheres could be great for biomolecular computing devices. These devices need to evolve in a way that allows them to respond to their environment.

## Supplementary Material



## Notes

Temporal resistance measurements of Fe­(NO_3_)_3_ solution over 60,000 s showing stable electrical behavior (mean = 0.150 MΩ, std = 0.062 MΩ) with minimal time-dependent variation, this establishes baseline Fe^3+^ ion properties for comparison with dynamic proteinoid–PS–Fe^3+^ hybrid systems (Figure S1); impedance magnitude analysis shows a 3-fold drop in electrical resistance, pure proteinoid systems have a resistance of about |*Z*| ≈ 0.20 MΩ, in contrast, proteinoid–PS hybrid systems show |*Z*| ≈ 0.07 MΩ, this change highlights better charge transfer and improved electrochemical performance in PS-templated bioelectronic assemblies (Figure S2); impedance distribution analysis reveals distinct electrical properties in pure proteinoid systems, these systems exhibit a wide scatter, with outliers reaching up to 3.4 MΩ, in contrast, proteinoid–PS hybrid systems display uniform, low-impedance clustering around ∼0.1 MΩ, this contrast highlights the templating effect of PS on enhancing electrochemical consistency (Figure S3); the temporal potential profile of Fe­(NO_3_)_3_ solution over 12,000 s shows no spontaneous oscillations, the potential steadily declines from +120 to −50 mV, then stabilizes around – 5 mV, this confirms that ferrous nitrate alone does not produce the bioelectronic activity observed in proteinoid-PS systems (Figure S4) (PDF)
