# Supplementary material for: Modulation of Proteinoid Electrical Spiking Activity with Magnetic Nanoparticles
Source: Langmuir. 2025 May 30;41(22):13974–92. doi: 10.1021/acs.langmuir.5c00932 (PMC12164352; doi:10.1021/acs.langmuir.5c00932)
Supplement: Supplementary file 1 [file la5c00932_si_001.pdf]

Supporting Information

Modulation of Proteinoid Electrical Spiking Activity with Magnetic Nanoparticles

Panagiotis Mougkogiannis[1,*] and Andrew Adamatzky[1]

[1]Unconventional Computing Laboratory, University of the West of England, Bristol, UK, BS16 1QY

Email: Panagiotis.Mougkogiannis@uwe.ac.uk

1 Electrical Characterization

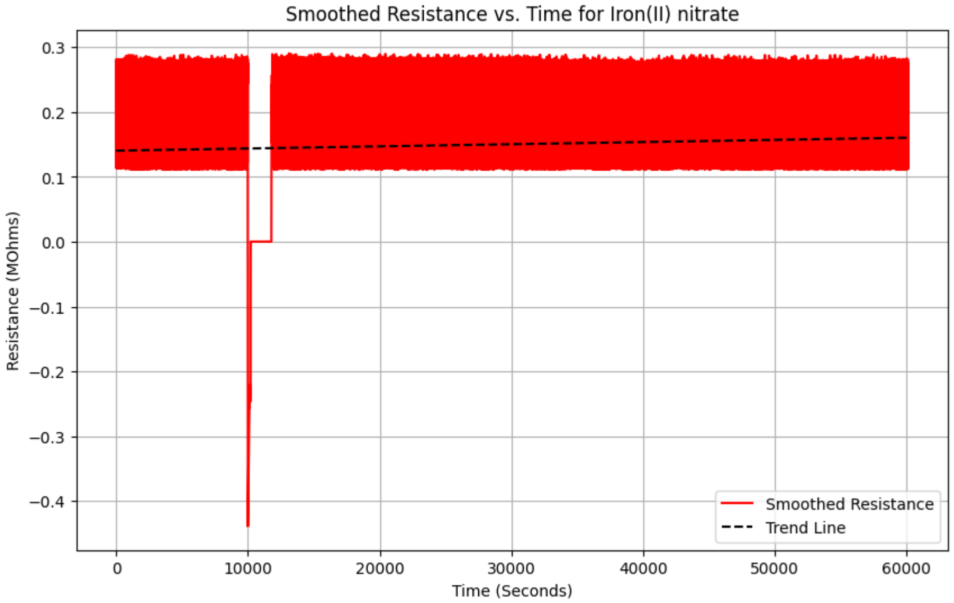

Figure S1: The $Fe(NO_3)_3$ solution shows a stable electrical profile over long measurement times. The smoothed resistance data (red solid line) remains around 0.15 MΩ for the full 60,000 seconds. It shows little change, with a standard deviation of 0.062 MΩ, and follows a steady trend line (black dashed line). The resistance values range from 0.12 MΩ to 0.29 MΩ, with the interquartile range (25th to 75th percentile) between 0.118 MΩ and 0.188 MΩ. This demonstrates that Fe^{3+} ions in water maintain stable electrical properties. There are no spontaneous changes in resistance or variations in conductivity over time. This stability provides important baseline data to contrast with the dynamic electrical behaviors observed in Fe^{3+}-PS:proteinoid hybrid systems. It confirms that the bioelectronic phenomena require the specific proteinoid microenvironment and cannot be attributed to the Fe^{3+} solution alone.

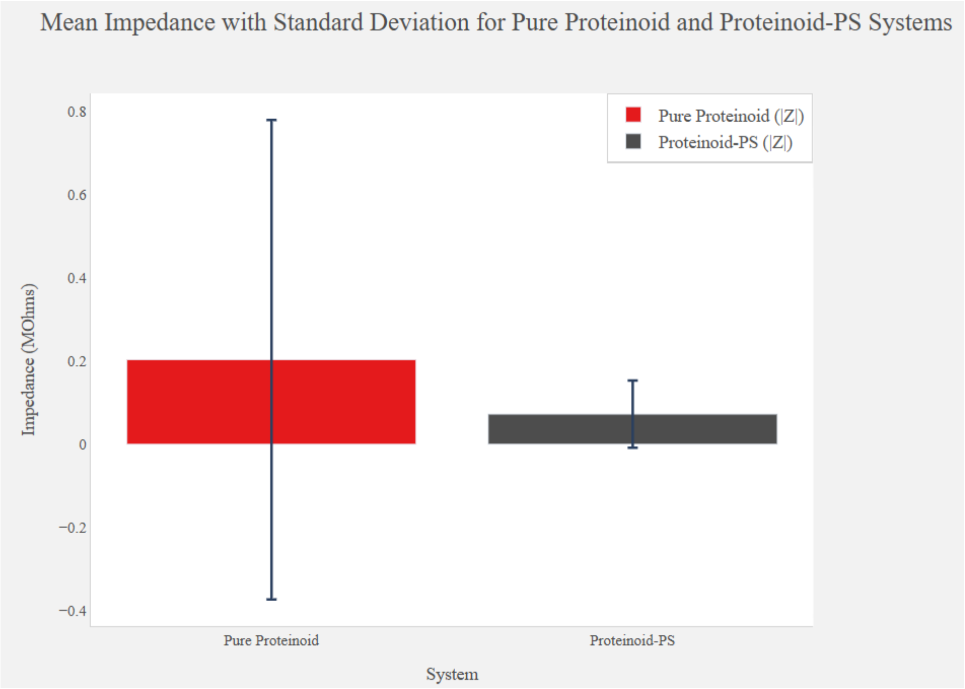

Figure S2: Impedance analysis shows that pure proteinoid systems conduct electricity less effectively than proteinoid-PS hybrids. The PS-modified variants demonstrate improved conductivity. Pure proteinoid systems (red bars) exhibit a higher mean impedance magnitude ($|Z| \approx 0.20$ MΩ) with substantial standard deviation, indicating variable charge transport properties across different microsphere assemblies. In contrast, proteinoid-PS hybrid systems (gray bars) demonstrate significantly reduced impedance ($|Z| \approx 0.07$ MΩ) with lower variability, reflecting more consistent and enhanced electrical conductivity. The addition of PS led to an approximately 3-fold decrease in impedance. This reduction indicates improved charge transfer and lower interfacial resistance. These improvements support the superior electrochemical performance observed in hybrid bioelectronic systems. Error bars represent the standard deviation across multiple measurements, highlighting how PS-templated proteinoid assembly enhances reproducibility.

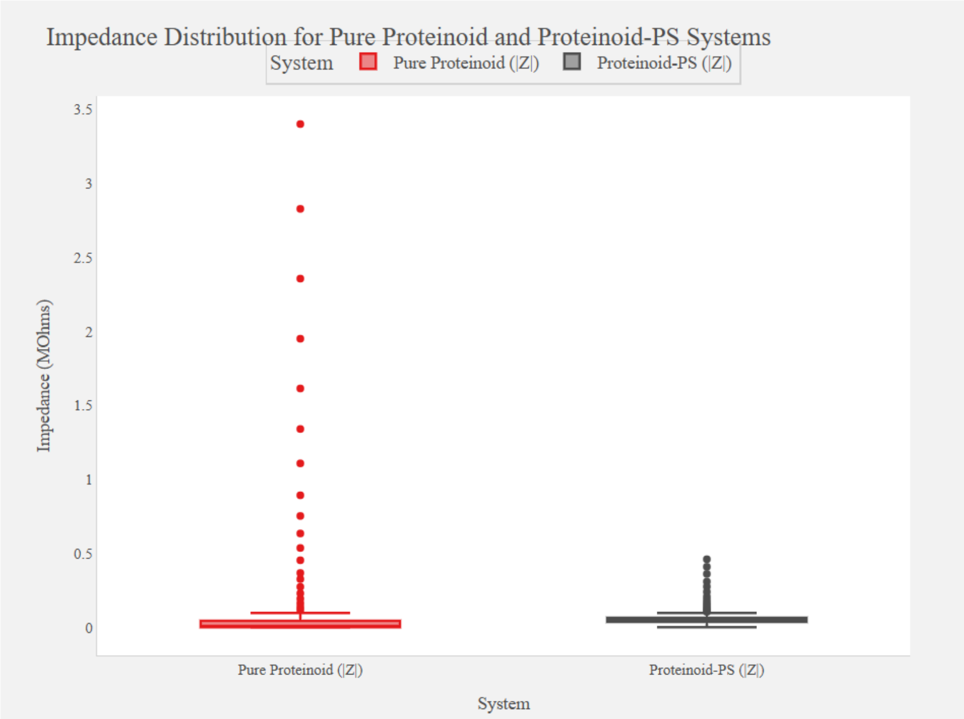

Figure S3: Impedance distribution analysis reveals distinct electrical behaviors between pure proteinoid systems and proteinoid–PS hybrid systems. Pure proteinoid systems (red) exhibit a wide range of impedance values, with many outliers extending up to 3.4 MΩ. This broad distribution suggests highly variable charge transport properties and indicates the presence of diverse electrochemical interfaces across different microsphere assemblies. The scattered data points, ranging from nearly 0 MΩ to several megaohms, underscore significant variability between individual systems. In contrast, proteinoid–PS hybrid systems (gray) demonstrate tight impedance clustering around 0.1 MΩ, with fewer outliers and reduced scatter. This implies that PS templating effectively standardizes electrical properties across the system. The substantial reduction in both mean impedance and variability highlights how PS incorporation improves uniformity and predictability in bioelectronic interfaces—an essential factor for reliable implementation in biomolecular computing applications.

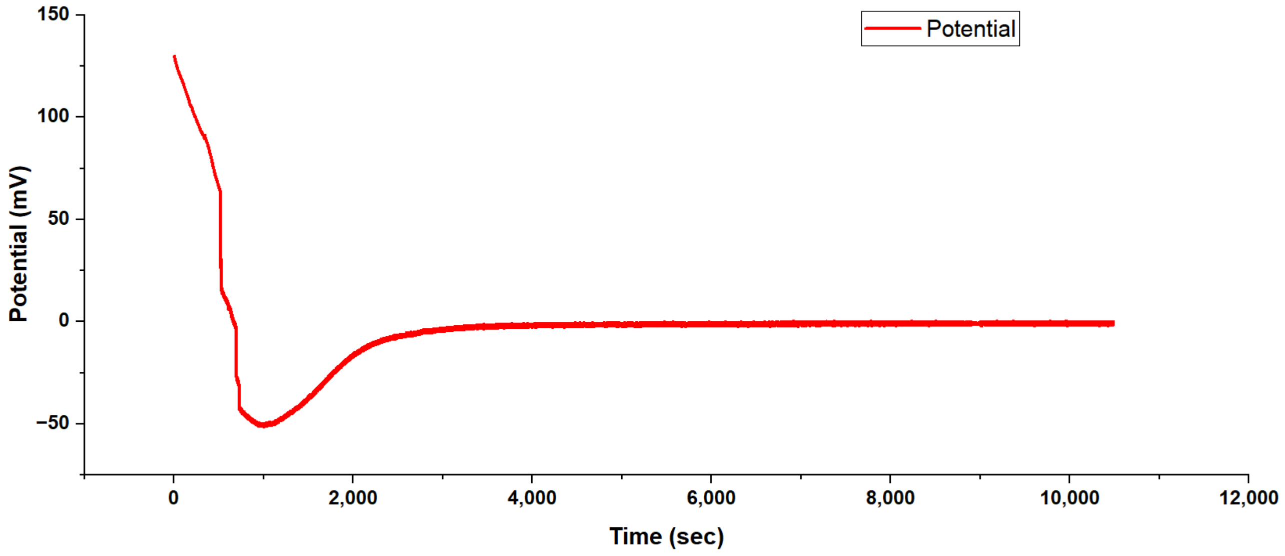

Figure S4: No spontaneous electrical oscillations were observed in the $Fe(NO_3)_3$ solution over a period of 12,000 seconds. The potential profile initially drops rapidly from $+120\,\mathrm{mV}$ to approximately $-50\,\mathrm{mV}$ within the first $1,500\,\mathrm{s}$, and then stabilizes around $-5\,\mathrm{mV}$ for the remainder of the observation period. The Fe^{3+} solution does not exhibit any spontaneous oscillations, periodic spikes, or rhythmic changes—features that are typically indicative of bioelectronic activity. The smooth, monotonic recovery curve confirms that ferrous nitrate solutions do not generate action potential-like signals, nor do they display the dynamic electrical patterns characteristic of proteinoid microsphere systems. This baseline behavior demonstrates that the complex oscillatory dynamics, membrane potential spikes, and computational logic observed in proteinoid–Fe^{3+} hybrid systems originate from proteinoid-mediated redox processes, and not from the intrinsic properties of Fe^{3+} ions in aqueous solution.
